# Prevalence and Dynamics of Ribosomal DNA Micro-heterogeneity Are Linked to Population History in Two Contrasting Yeast Species

**DOI:** 10.1038/srep28555

**Published:** 2016-06-27

**Authors:** Stephen A. James, Claire West, Robert P. Davey, Jo Dicks, Ian N. Roberts

**Affiliations:** 1 National Collection of Yeast Cultures, Institute of Food Research, Norwich Research Park, Norwich, NR4 7UA, UK; 2 Digital Biology, Earlham Institute, Norwich Research Park, Norwich, NR4 7UH, UK

## Abstract

Despite the considerable number and taxonomic breadth of past and current genome sequencing projects, many of which necessarily encompass the ribosomal DNA, detailed information on the prevalence and evolutionary significance of sequence variation in this ubiquitous genomic region are severely lacking. Here, we attempt to address this issue in two closely related yet contrasting yeast species, the baker’s yeast *Saccharomyces cerevisiae* and the wild yeast *Saccharomyces paradoxus*. By drawing on existing datasets from the Saccharomyces Genome Resequencing Project, we identify a rich seam of ribosomal DNA sequence variation, characterising 1,068 and 970 polymorphisms in 34 *S. cerevisiae* and 26 *S. paradoxus* strains respectively. We discover the two species sets exhibit distinct mutational profiles. Furthermore, we show for the first time that unresolved rDNA sequence variation resulting from imperfect concerted evolution of the ribosomal DNA region follows a U-shaped allele frequency distribution in each species, similar to loci that evolve under non-concerted mechanisms but arising through rather different evolutionary processes. Finally, we link differences between the shapes of these allele frequency distributions to the two species’ contrasting population histories.

Recent years have seen the debate on the prevalence, indeed the existence, of ribosomal DNA (rDNA) sequence heterogeneity intensify. On the one hand, systematists exploiting the valuable properties of the Internal Transcribed Spacer (ITS, a sub-region of the rDNA) for phylogenetic inference^1^ have observed intra-individual rDNA sequence variation for decades^2^,^3^,^4^. On the other hand, a recent genome sequencing study has suggested only low levels of sequence heterogenity exist in the rDNA region^5^.

The rDNA, a key component of an organism’s essential protein-making machinery and therefore common to species spanning the tree of life, is arranged at one or more genomic loci. While prokaryotes and archaea package their rDNA in operon or operon-like structures^6^,^7^, in eukaryotes these loci comprise tandem arrays of rDNA units long thought by many to be homogeneous in sequence. For example, in the baker’s yeast *Saccharomyces cerevisiae* ~150 rDNA units of 9.1 kb in length are found in tandem at a single locus on chromosome XII^8^. Despite individual unit sequences being subject to evolutionary events such as point mutation, homogeneity along an entire array may be reinstated through concerted evolutionary processes such as unequal sister chromatid exchange and gene conversion^9^. This balance of heterogeneity-inducing mutations and homogeneity-promoting concerted evolutionary events is key to understanding micro-heterogeneity in this important genomic region. Taking a snapshot of the rDNA at any one point in time shows the nature of this balance at that moment.

Whole genome sequencing and re-sequencing projects provide the means to take such a snapshot and to characterise fully both intra- and inter-genomic rDNA sequence variation across a range of taxonomic scales, within and between the kingdoms of life. These approaches have the power to overcome sampling biases inherent to, for example, PCR-based techniques. In this study we aim to exploit an existing genome re-sequencing resource in order to finely characterise rDNA sequence variation in multiple strains of two closely related yet contrasting yeast species, *S. cerevisiae* and its wild relative *Saccharomyces paradoxus*.

We build upon recent examinations of rDNA sequence variation in relevant genome sequences. For example, Ganley and Kobayashi^5^ analysed Whole Genome Shotgun Sequencing (WGSS) data ranging between 1.7 and 7.7× coverage for five fungal genomes. Here, the authors identified just 13 high-stringency polymorphisms in *S. paradoxus* and 4 in *S. cerevisiae*, in line with the concept of rDNA sequence homogeneity. Simon and Weiß^10^ examined rDNA polymorphism in 4 plant pathogens at 3 sub-regions of the rDNA unit, discovering higher nucleotide diversity levels than Ganley and Kobayashi, notably encompassing the one species common to their analyses (*Aspergillus nidulans*). In a previous study^11^, we examined the rDNA regions of 34 strains of the baker’s yeast *S. cerevisiae* from WGSS data generated within the Saccharomyces Genome Resequencing Project^12^. Due to the greater breadth of our strain sampling than in prior studies, we were able to report high levels of sequence variation amongst individual rDNA units, ranging from 10 to 76 polymorphisms per strain across 227 variable sites. Many of the polymorphisms detected in this analysis were not fully resolved, in that for a particular strain they did not occur in all repeats of the rDNA array, and so could not be regarded as conventional single nucleotide polymorphisms (SNPs). The term partial single nucleotide polymorphism, or pSNP, was introduced for this type of intra-genomic sequence variation, in essence describing one aspect of an organism’s deviation from a perfect concerted evolutionary process. A further interesting finding from this study was an association between the number of pSNPs present in an rDNA array and the degree of genome mosaicism observed. The level of genome mosaicism attributed to each strain was denoted as either Structured or Mosaic, according to population structure analyses carried out within the first major population genetic study of yeast^13^. In that study, the Structured strains were believed to have been subject to a lower - but not necessarily negligible - degree of hybridisation than the Mosaic strains in their evolutionary history.

Most recently, we conducted a reanalysis of this *S. cerevisiae* dataset, alongside that of 26 SGRP strains of *S. paradoxus*^14^. Using the TURNIP software^15^ for SNP and pSNP variation discovery we showed, for both species, that the phylogenetic signal contained within these rDNA-based variants was consistent with that found in genome-wide SNP datasets. Furthermore, we refined the categorisation of genome mosaicism to include three levels: Structured clean, Structured mosaic and Mosaic. This subdivision of the former Structured strains into two groups followed the observation that levels of genome hybridisation were believed to be minimal for strains classified at Structured clean whereas they were thought to be moderate for Structured mosaic strains, though still less than strains classified as Mosaic.

Here, we delve further into this two-species dataset, identifying a wealth of inter-genomic variants while also uncovering and describing a wider range of intra-genomic rDNA sequence variation, all at an unprecedented level of discrimination. We show that *S. cerevisiae* and *S. paradoxus* both possess high levels of sequence heterogenetity within their respective rDNA loci yet exhibit rather different mutational profiles. We characterise, for the first time, unresolved insertion and deletion polymorphisms in the rDNA arrays of both species, and introduce the terms pINS and pDEL to describe these intra-genomic polymorphism types respectively. We further suggest that pINS and pDEL polymorphisms may each be further characterised into two subtypes. We discover that patterns of pINS and pDEL polymorphisms, like pSNPs, are an indicator of genome mosaicism. We show for the first time that for strains within each species set, allele frequencies of unresolved polymorphisms exhibit U-shaped distributions, such as do loci evolving under non-concerted mechanisms, and that the shapes of these distributions relate to the species’ population history. Finally, we discuss how new mathematical models of rDNA evolution, when fitted to these and other similar datasets, could vastly increase our current understanding of concerted evolutionary processes across a wide range of organisms.

## Results

### rDNA sequence variation is abundant in both species

Our use of the TURNIP software for variation discovery enabled us to uncover a wide range of polymorphisms in both species. In addition to fixed polymorphisms, manifested as variants present in all rDNA units within a strain, unresolved polymorphisms, instead present in only a subset of rDNA units, were also identified. Whilst we have previously categorised and analysed partial single nucleotide polymorphisms (pSNPs)^11^,^14^, here we introduce partial insertions and partial deletions along with their respective abbreviations, pINSs and pDELs. We also recorded a small number of cases where distinct polymorphism classes coincided at the same sequence site within different rDNA units of the same strain, and which we term complex mutations (CX). In summary therefore, our TURNIP analysis identified three classes of fixed, inter-genomic variants (SNPs, insertions, deletions) and their respective partial, intra-genomic forms (pSNPs, pINSs, pDELs), along with complex mutations that could involve combinations of two or more of these six polymorphism types.

Tables 1 and 2 present a breakdown of the sequence variants (type and total) found in each strain of *S. paradoxus* and *S. cerevisiae* respectively. Overall, 970 polymorphisms were identified across the entire set of 26 *S. paradoxus* strains and 1,068 across the 34 *S. cerevisiae* strains. The levels of sequence variation (fixed and partial variants combined) identified in the rDNA arrays of the 60 strains examined were found to vary markedly, in *S. paradoxus* ranging from a single polymorphism in the European strain Q89.8 to 114 in the American strain DBVPG6304, and in *S. cerevisiae* from 4 polymorphisms in the W303 strain to 49 in YS4 and DBVPG1853 (see Supplementary Table S1 for strain information for both species and Supplementary Table S2 for details of variants).

#### Fixed polymorphisms

**Figure 1:**
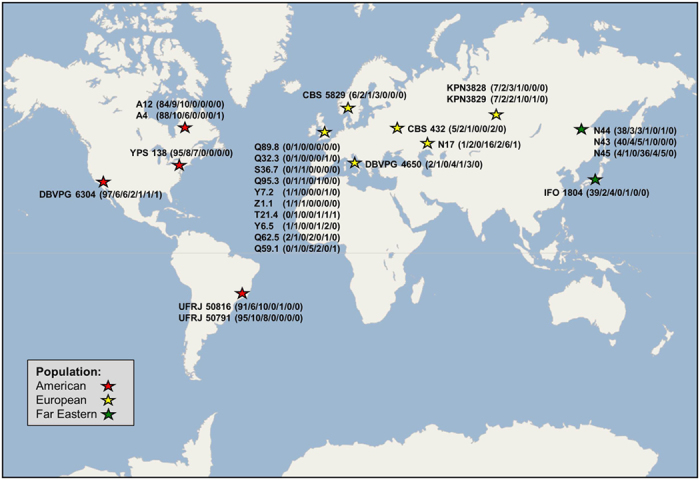
Geographical Location of the *S. paradoxus* Strain Collection. World map with the location of the collection sites for the *S. paradoxus* strains indicated by stars. Stars are coloured by population type. In brackets following each strain are the numbers of SNPs, insertions, deletions, pSNPs, partial insertions, partial deletions and complex mutations identified for that strain in this study. World map template downloaded and modified from https://commons.wikimedia.org/wiki/File:World_map_blank_shorelines_semiwikimapia.svg.

This class of variant is key to understanding the inter-relationships amongst the strains under analysis, being proportional to the genetic divergence between them. Of the 970 polymorphisms identified within the *S. paradoxus* strains (see Table 1), 853 (87.9%) were found to be fixed, with SNPs clearly the largest contributor to this group (82.6% of fixed polymorphisms). Compared to CBS 432, the species type strain and European *S. paradoxus* reference strain, the 16 European strains were found to possess the fewest number of fixed polymorphisms (64; 4.0 per strain). The four Far Eastern strains exhibited almost nine times as many polymorphisms per strain (143; 35.8 per strain), and the six American strains were the most diverse group with almost twenty seven times as many polymorphisms per strain (646; 107.7 per strain), the polymorphism number clearly growing with geographic divergence from the reference strain (Fig. 1). The major exception to this trend was the Far Eastern strain N-45, which possessed just 5 fixed polymorphisms, a number more akin to strains within the European group.

We also identified 569 fixed polymorphisms in the 34 *S. cerevisiae* strains (see Table 2), comprising only just over half (53.3%) of the 1068 variants observed in this species. While SNPs were again the most prevalent fixed variant (59.6%), deletions were also frequently observed within this species (37.8%). As expected from their complex genome structure, the 15 mosaic strains were found to possess the fewest number of fixed polymorphisms (159; 10.6 per strain). Fixed variants were found to be slightly more prevalent in the 12 structured mosaic strains (200; 16.7 per strain) and most frequent in the 7 structured clean strains (210; 30 per strain). The clearest exception to this trend was the Structured mosaic strain K11, which was found to possess 37 fixed polymorphisms, the second highest number for all strains in this analysis.

#### Partial polymorphisms

This class of variant provides an indication of the distance from a state of complete sequence homogeneity amongst a strain’s rDNA units. We believe this distance to be the result of a complex interplay of factors such as the degree of genome hybridisation, rDNA copy number and genomic ploidy (see Discussion for more on these points). While the various concerted evolutionary processes that act on the rDNA array may lead to eventual fixation or loss of a partial polymorphsim, we showed recently that pSNPs are found in the rDNA array of both *S. cerevisiae* and *S. paradoxus* strains^14^ and that they provide information on genetic divergence that may be used in combination with that provided by fixed polymorphisms.

**Figure 2:**
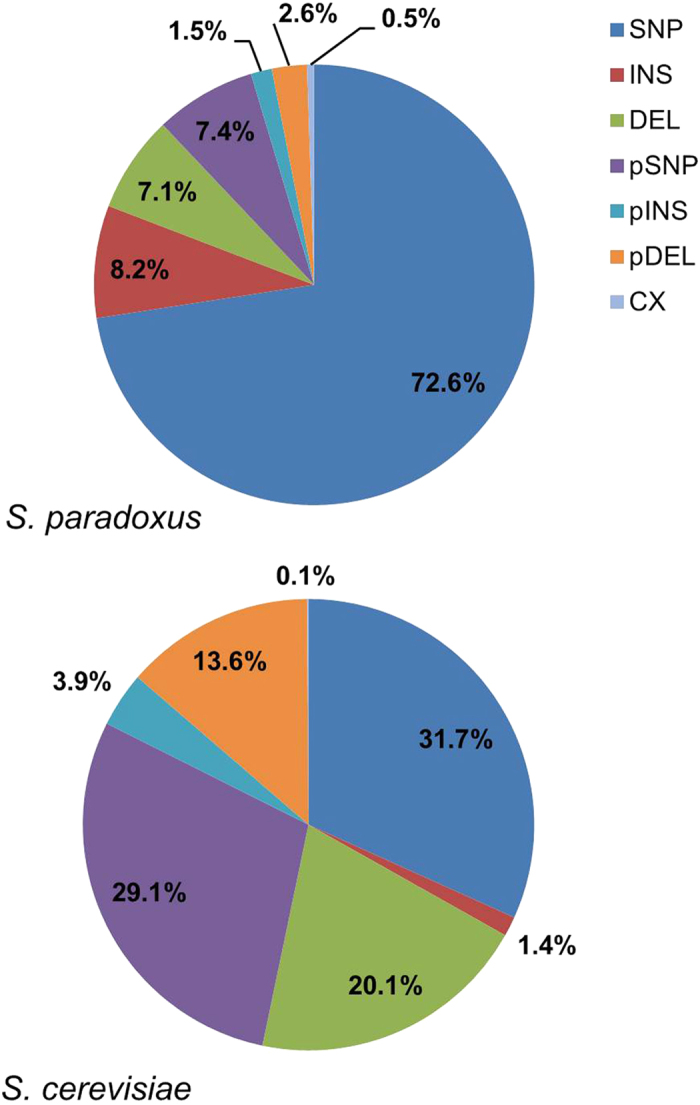
Mutational Profiles of *S. paradoxus* and *S. cerevisiae*. Percentages of each type of mutation within each species are shown, clearly indicating contrasting mutational profiles between the two species.

In *S. paradoxus*, pSNPs tend to be possessed in modest numbers (Table 1; an average of 2.8 pSNPs per strain) with occasional high frequencies, as for strains N-17 (European strain; 22.2% of all pSNPs) and N-45 (Far Eastern strain; 50.0% pSNPs), a potential indicator of a genome hybridisation event. In contrast, pSNPs are generally more prevalent amongst *S. cerevisiae* strains (Table 2, Fig. 2; an average of 9.1 pSNPs per strain), most notably in industrial strains, with their greater numbers hypothesised to be a feature of a higher level of hybridisation between strains of that species.

This study has also shown that unresolved insertions (pINS) and deletions (pDELs) are a feature of the variation possessed by both species. It is interesting to observe, for example, that unresolved insertions are more frequent than fixed insertions in *S. cerevisiae*. We also noted earlier the prevalence of deletions in *S. cerevisiae* rDNA arrays when compared to *S. paradoxus* (Fig. 2). A closer inspection reveals the majority of *S. cerevisiae* deletions (61.7%) are found in the non-coding IGS1 region, with the majority of IGS1 deletions specific to just five small regions, all of which are homopolymeric poly(dA).poly(dT) tracts (see Supplementary Figure S1 for an example). Approximately 40% of all deletions in *S. cerevisiae* are unresolved. We can further characterise these unresolved deletions into two types. Type 1 pDELs correspond to tracts of a fixed size that are found to be deleted in a subset of rDNA array units. Type 2 pDELs are more complex, with tracts also varying in size between the units in which they have been deleted. Examples of Type 1 and Type 2 pINS and pDEL variants are described in more detail in Supplementary Figure S1. Long homopolymeric tracts, particularly poly(dA).poly(dT) tracts, are known to be unstable and prone to slip-strand replication errors, which in turn can give rise to tract length variation^16^. While Ganley and Kobayashi^5^ noted that a high number of deletions may be indicative of genome size reduction^17^, the limited size and scope of the rDNA deletions means we are likely observing a different phenomenon. The relative lack of insertions in homopolymeric tracts is also puzzling, though the overall lack of such events in all regions of the rDNA suggest this may be a general mutational preference in *S. cerevisiae* strains, the reason for which is currently unclear.

Partial insertion events may also be characterised in a similar way, with both identical and non-identical insertion sequences noted for distinct pINSs. Type 2 mutations are, in general, difficult to estimate as they require both an alignment to a reference sequence, which is analysed site-by-site, and examination of the raw sequencing reads underpinning the alignment for precise estimation, as opposed to examination of just an alignment for other types of mutation. Without explicit software coding for such eventualities, accurate manual estimation would be overly costly in a high-throughput analysis. Given that such functionality has yet to be added to the TURNIP software, the numbers of Type 1 and Type 2 pINSs and pDELs in each of the two species, as shown in Table 3 and which are estimated from the alignment alone, should be considered to be a rough estimate. It is notable that Type 2 mutations, whether partial insertions or partial deletions, appear to be proportionately less frequent in *S. paradoxus* than in *S. cerevisiae*.

Further examination of the distribution of pINSs and pDELs across the strains of the two species shows that, as for pSNPs but arguably to a lesser extent, they are indicators of putative hybridisation events. For example, in *S. paradoxus* we see that pDELs are most prevalent in N-17 and N-45, our two putative hybrid strains, and that pINSs are most frequent in N-45. Furthermore, in *S. cerevisiae*, 32 out of 42 pINSs and 98 out of 145 pDELs are seen in strains classified as mosaic, with only 1 pINS and 10 pDELs identified in structured clean strains. Calculating Pearson’s correlation coefficients between the number of pSNPs and the combined number of pINSs and pDELs in each species gives *r* = 0.867 and *r* = 0.896 in *S. paradoxus* and *S. cerevisiae* respectively, strongly supporting the observation that strains high in one type of intra-genomic variant are likely to be high in another.

### Contrasting mutational profiles exist between species

Comparing the mutational profiles of the two species groups (Fig. 2; i.e. the proportions of each of the seven mutation types) indicates that single nucleotide polymorphisms, whether fixed as SNPs or unresolved as pSNPs, were more prevalent in *S. paradoxus* than in *S. cerevisiae* (80.0% compared with 60.9%). Furthermore, the balance of these two polymorphism types was very different between the two species, with approximate ratios of 10:1 and 1:1 SNPs to pSNPs in *S. paradoxus* and *S. cerevisiae* respectively.

Our new analysis has further uncovered significant numbers of insertion and deletion polymorphisms, not considered in our previous studies^11^,^14^. These mutations account for almost 30% of all the variation detected in the two *Saccharomyces* species. However, one of the striking differences between the variation identified in the two species (Fig. 2) is the large number of deletions found in *S. cerevisiae*, with 33.7% of all variation attributed to deletions (fixed or partial), compared to only 9.7% in *S. paradoxus*. In contrast, insertion mutations (again, both fixed or partial) though generally more rare are more prevalent in *S. paradoxus*, contributing to 9.8% of all variation in that species compared to 5.3% in *S. cerevisiae*. Overall, a comparison of the proportions of fixed variants (i.e. SNPs, INSs and DELs) in *S. cerevisiae* (0.533) versus *S. paradoxus* (0.879) strains gives a *p*-value < 2.2 *e*^−16^ in an independent two-sample t-test, strongly supporting a difference between the two populations.

In general, the mutational profiles show that certain types of polymorphism are favoured in each species and furthermore that these differ between species. In their earlier analysis, Ganley and Kobayashi^5^ also found a biased mutational profile in *S. paradoxus*, though they were not able to establish one in *S. cerevisiae* due to a lack of identified mutations.

### Sites of variation within the rDNA unit sequence are non-random

**Figure 3:**
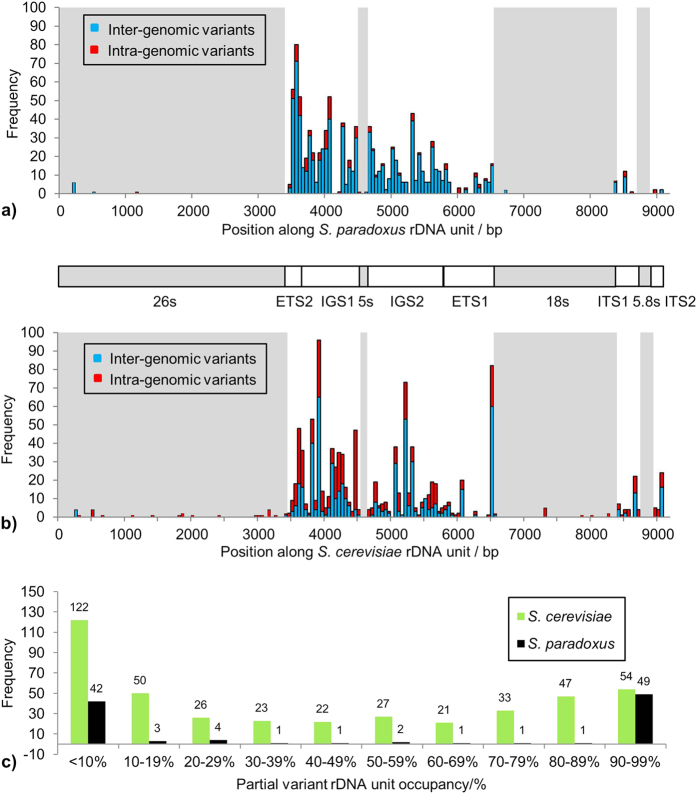
Distribution of Sequence Variation Along the rDNA Unit. The distribution of rDNA sequence variants within the rDNA unit and their unit occupancy frequencies along the tandem array. (**a**) Inter- and intra-genomic variants within the *S. paradoxus* dataset; fixed, inter-genomic variants (SNPs + INSs + DELs) are shown as blue bars while partial, intra-genomic variants (pSNPs + pINSs + pDELs) along with complex variants are shown as red bars, with the boxed areas in light grey highlighting coding RNA regions. Representation of an rDNA unit is shown below. Most variation is identified in the ETS2, IGS1 and IGS2 regions. (**b**) Inter- and intra-genomic variants within the *S. cerevisiae* dataset; data represented as in (**a**). Most variation is identified in the IGS1, IGS2 and ETS1 regions. (**c**) Bar chart showing unit occupancies of most intra-genomic variants (pSNPs + Type 1 pINSs + Type 1 pDELs) in the *S. paradoxus* and *S. cerevisiae* datasets, in unit occupancy bins of size 10%. Though both exhibiting U-shaped curves, the variant occupancy distributions for *S. cerevisiae* and *S. paradoxus* are markedly different.

In addition to the varying levels of sequence variation found in the individual rDNA datasets of both species, the detected variation was found to be distributed unevenly over the rDNA repeat (Table 4). Figures 3a and 3b depict the locations of both fixed and partial variants identified in *S. paradoxus* and *S. cerevisiae* respectively. For both species, most of the identified polymorphisms were discovered in the non-coding ETS2, IGS1, IGS2 and ETS1 regions. High numbers of polymorphisms were observed for both species within IGS1 (45.5% in *S. cerevisiae* and 39.7% in *S. paradoxus*) and IGS2 (30.2% in *S. cerevisiae* and 35.9% in *S. paradoxus*). However, *S. paradoxus* strains appeared to favour mutations within ETS2 (14.8%) over ETS1 (6.0%), with the opposite situation in *S. cerevisiae* (3.5% in ETS2 versus 10.5% in ETS1). Furthermore, the mutation types contributing to these regional proportions differed markedly between species (e.g. the most prevalent IGS1 mutations in *S. paradoxus* were SNPs but in *S. cerevisiae* were deletions).

In contrast, and perhaps not surprisingly in view of functional constraints, very few mutations were found in the rRNA-encoding genes. For instance, no variants were detected in the highly conserved 5S or 5.8S rRNA genes in *S. cerevisiae* strains, while just a single partial deletion was observed in the 5S region of the putative hybrid N-17 *S. paradoxus* strain. The 26S and 18S rRNA genes appeared slightly more tolerant to mutation, with 33 and 12 variants across the two species respectively, all but four of which were SNPs or pSNPs. Interestingly, in both species pSNPs in coding regions are observed at low frequency, consistent with Ganley and Kobayashi’s idea of a tolerance threshold^5^, where a small number of units within the rDNA array may harbour a polymorphism without detrimental effect.

We carried out two-sample t-tests between the proportions of variants in each species, in each of the ten rDNA regions, correcting for the false discovery rate of 0.05 using the Benjamini-Hochberg procedure. The results (*p* < 2.2 *e*^−16^, *p* = 6.141 *e*^−6^, *p* = 0.0002375, *p* = 0.006765, *p* = 0.006883, *p* = 0.008046, *p* = 0.01406, *p* = 0.03142, *p* = 0.2939 and *p* = *NaN* for ETS2, ITS2, ETS1, 26S, IGS2, IGS1, ITS1, 18S, 5S and 5.8S respectively) rejected the supposition of no species-related difference in variant proportions for all rDNA regions except for 5S and 5.8S, unsurprising given the paucity of variants in these two regions.

### Partial variant allele frequencies follow a U-shaped distribution

Plotting the intra-genomic rDNA unit occupancy frequencies of partial variants (though Type 1 variants only for pINSs and pDELs, due to the difficulty in estimating such frequencies for Type 2 variants), which can be considered as allele frequencies and which are estimated by the percentage of variant reads per strain, shows an interesting pattern (Fig. 3c). For both *S. paradoxus* and *S. cerevisiae* datasets the distribution follows a U-shaped curve, though the *S. cerevisiae* distribution is much flatter. U-shaped distributions are often observed in biological datasets, including both allele frequency^18^ and gene frequency^19^ datasets within populations, as predicted by mutation-drift theory. Beta distributions may be fitted to such datasets to help characterise and compare them. Fitting beta distributions, using the R statistical environment, to the pSNP/SNP datasets shown in Fig. 3c gave shape parameter Maximum Likelihood Estimates of *α* = 0.375, *β* = 0.371 with standard errors of (0.046, 0.046) for *S. paradoxus* and *α* = 0.635, *β* = 0.818 with standard errors of (0.039, 0.053) for *S. cerevisiae*, formalising the observed differences between them.

The estimated unit occupancies, or proportion of units within an rDNA array estimated to harbour a particular variant, were plotted for pSNP, pINS and pDEL variants individually (again Type 1 variants only for pINSs and pDELs) in each of the two species datasets. When examining such allele frequency plots it is important to realise that a 10% occupancy is equivalent to a 90% occupancy, where each value indicates a 10% divergence from fixation of one of two allelic forms (and more generally, an *x*% occupancy is equivalent to a (100 − *x*)% occupancy). Five of the six plots revealed U-shaped distributions (Supplementary Figure S2), the single exception being pINSs in *S. cerevisiae*, whose right-handed peak lies in the occupancy bin 70–79% rather than the expected 90–99%. The two pSNP curves were both strongly U-shaped, unsurprising considering they formed the largest sub-section of the combined distribution and were therefore likely to be less susceptible to noise. The *S. paradoxus* pSNPs can effectively be subdivided into two categories, 29 with very low occupancy (<10%), and 38 with very high occupancy (>90%). Only 5 of the 72 *S. paradoxus* pSNPs (6.9%) were found to have an intermediate occupancy, varying only between 28.8% and 43.6%. In contrast, 183 of the 311 *S. cerevisiae* pSNPs (58.8%) possessed an occupancy of between 10% and 90%, spanning the entire range of values.

Although some features of pSNP/SNP variation, such as their quantity and location, can readily be related to characteristics of their harbouring species, others are less obvious without a deeper understanding of strain origins and inter-relationships. For example, although the overall pSNP frequency distribution for the *S. cerevisiae* strains is characteristically shallow, this shape is only retained for the Mosiac and Structured mosaic strain subsets (Supplementary Figure S3). In constrast, the pSNP distribution of the Structured clean strains shows a similar occupancy pattern to the *S. paradoxus* dataset, potentially the consequence of a common low incidence of genome hybridisation. It is interesting to note that the majority of the intermediate occupancy pSNPs in the Mosaic and Structured mosaic datasets are found in the Wine/European and Sake groups. As the majority of these strains are from a fermentation origin, we hypothesise they are more likely to have undergone hybridisation (with other fermentation strains) than their wilder cousins from the Malaysian, North American and West African groups, which possess pSNPs only at very low or very high occupancy.

### Sequence read coverage is consistently high in both species

The sequence read coverage of each strain set, calculated by mapping reads to 20 bp target windows of the relevant rDNA consensus sequence, was determined to ensure our results were both supported by adequate read depth and not affected significantly by biased read location. Detailed coverage plots were produced and are shown and discussed in Supplementary Figure S4. Overall, we found the vast majority of rDNA sequence sites to be covered at good read depth in all strains in both species, lending support to the results achieved above.

## Discussion

We have conducted the most detailed analysis of rDNA sequence variation to date. Within each species, we have uncovered sequence variation that is both substantial in quantity and rich in evolutionary information. Unlike many preceding studies, our analysis has encapsulated variation across the whole rDNA unit, rather than sub-regions within it, and has examined a broader range of polymorphism types across a larger number of genomes. In particular, variants found to be unresolved across an rDNA array, and which were previously undefined, have now been formalised as partial insertions (pINSs) and deletions (pDELs), and even subcategories of such variants have been brought to notice for the first time.

The population histories of *S. paradoxus* and *S. cerevisiae* are believed to differ greatly. In particular, a high frequency of genome mosaicism has previously been inferred in one species, *S. cerevisiae*, but not the other, *S. paradoxus*^13^. It is compelling to speculate that the hybrid, mosaic strains in *S. cerevisiae* may be linked to human traffic and/or industrial processes whereas they are considerably less likely to arise in the wild *S. paradoxus*. For such contrasting species, we might expect to see major differences in features of their rDNA variation, such as relative proportions of mutation types. Indeed, we have shown in this study that the mutational profiles of the two species are highly different, and even how some mutation types are more prevalent within certain sub-regions of the rDNA array. Furthermore, we have seen that the dearth of rDNA variants within coding regions of the rDNA unit, first observed in *S. cerevisiae*^11^, also extends to *S. paradoxus* and is likely a general feature of rDNA variation. Given the much higher frequencies of variants in the remaining regions, we speculate that purifying selection must act strongly on the rRNA gene sequences to maintain this pattern. Only ~50% of rDNA units are transcriptionally accessible due to their heterochromatin state, and only a fraction of the remainder are active at any point in time^20^. In such a situation, even deleterious partial mutations with low frequencies could perhaps be tolerated given their limited transcription, with only mutations with higher frequencies selected against. An exception to this scenario might be in cases where additional mutations that maintain the RNA hairpin structure are also present. It will be interesting to see whether we can formerly test such hypotheses with real datasets in future.

Comparisons within mutation types can also be illuminating. In *S. paradoxus*, we observe the vast majority of pSNPs to have variant occupancies of less than 10% or greater than 90%, and SNP variation is high within the species, suggesting that many previous pSNPs have become fixed. In contrast, over half of all identified pSNPs were found to have occupancies within the 10% to 90% range in *S. cerevisiae*, and the numbers of pSNPs and SNPs were considerably higher and lower respectively than those seen in *S. paradoxus*. We have also shown the numbers of pINS and pDEL variants to be strongly correlated with the numbers of pSNPs in both species, suggesting that these variants may also be indicators of genome mosaicism, a discovery that warrants further investigation. Furthermore, we have shown for the first time that the unit occupancy values of rDNA variants, which can be considered to be allele frequencies, follow a U-shaped distribution within each dataset. While U-shaped distributions have been identified within populations for single copy loci for some time, the concerted evolutionary processes that led to those observed here are very different from those responsible for the single copy loci distributions.

Frequencies of variants across the rDNA array are likely to be affected by the number of copies of the rDNA unit and the hybridisation history of the strain. Furthermore, the number of rDNA loci within a genome and its ploidy may also be factors in our ability to determine variant frequencies, since if we cannot discriminate between different loci or sub-genomes, we will be merely averaging variant frequencies across loci/sub-genomes within a strain. In a recent study^14^, we estimated the rDNA copy numbers of all 60 strains included within this analysis. The average copy number across the 26 *S. paradoxus* strains is much smaller than that of the 34 *S. cerevisiae* strains, at 69 and 99 respectively. Furthermore, we believe that hybridisation has been a less significant phenomenon in the evolution of *S. paradoxus* than of *S. cerevisiae*^13^. Although the fairly low genome coverage of the strains analysed here prevents us from computationally estimating their ploidy, we strongly believe that all 60 strains possess a single rDNA locus. Consequently, we hypothesise that copy number and hybridisation history are the major factors underpinning the observed variant distributions uncovered in this analysis and we look forward to testing this hypothesis formally in future studies. In particular, we aim to understand how the differences in the U-shaped variant frequency distributions observed for the two species arose. U-shaped distributions are a pervasive feature of biological datasets, predicted under mutation-drift evolutionary theory. Computer simulations show that ‘steep’ U-shaped distributions, such at those seen for *S. paradoxus*, can form quickly from initial mutations [ref. 21, Chapter 5], whereas the analogous distributions of *S. cerevisiae* are considerably flatter. Within our future analyses, we aim to understand in detail the roles of rDNA copy number and hybridisation events on the establishment of these distributions and how ploidy and rDNA locus number may confound their delineation.

Comprehensive characterisation of rDNA-based variation naturally relies on our ability to sequence the whole rDNA locus (or loci). At present, we are not able to assemble rDNA sequences into ordered tandemly arranged units, due to their highly repetitive nature. A consequence of this is that we cannot be sure that our target sequence has been uniformly sampled across all of its tandem copies, and hence some variation may still be missing. However, current sequencing technologies, where we can deeply sample sequences of interest, suggest that we are approaching a complete coverage of the entire locus. Furthermore, promised technological advances, particularly those in the area of long read sequencing, mean that assembly of repetitive sequences may soon be possible. Consequently, we can be confident that with current approaches, we can make good estimates of rDNA sequence variation and that these will improve further in the near future. Furthermore, more sophisticated algorithms, particularly those employing probabilistic strategies, will be helpful for calling variants in larger strain sets. In particular, a finer characterisation of Type 2 partial insertions and deletions, which optimally require automated examination of reads in addition to read alignments, may be achieved by improved variant calling methods.

The datasets developed and analysed here offer a fascinating snapshot of concerted evolution in action. Many groups, including us, are interested in learning more about these concerted evolutionary processes by modelling them mathematically. An important recent study^22^ used experimental approaches to monitor the gain and loss of introduced mutations within the *S. cerevisiae* rDNA array, discovering important characteristics of the spatial and temporal dynamics of the concerted evolutionary process within this organism. The knowledge and datasets we have developed here are a further step towards achieving successful models that can fully exploit the rich source of evolutionary knowledge held within the rDNA array. For example, beta distribution parameter estimates derived from partial variants may be particularly useful in modelling the spread of allele frequencies by processes such as unequal sister chromatid exchange and gene conversion, determining the balance of such processes in a range of organism groups, and discovering how the spread of mutations along an rDNA array may be constrained by genome hybridisation. Furthermore, rDNA is now known to play a key role in cancer cell proliferation^23^, and future studies will focus on hunting for rDNA sequence or copy number variants associated with disease states. Given the ubiquity of the rDNA genomic region, the prospect of using mathematical models to analyse the genomes of a wide range of species, and optimised variant-hunting methods for associations with genetic disease, is an attractive one.

## Methods

### Data Preparation

Raw WGSS reads were obtained, in FASTQ format, from the SGRP *S. paradoxus* and *S. cerevisiae* datasets^12^. Twenty six *S. paradoxus* and thirty four *S. cerevisiae* strains formed the overall dataset, a total of 716,316 and 703,641 sequencing reads respectively. The rDNA consensus sequence for *S. paradoxus* was obtained from GenBank [ref. 24, Accession Number:BR000309.1] for the type strain NRRL Y-17217, also known as CBS 432^5^, a sequence of 9,103 bp in length. The *S. cerevisiae* consensus sequence was obtained for the reference strain S288c from the Saccharomyces Genome Database^25^, a sequence of 9,137 bp in length.

The TURNIP software (version 1.3) for analysis of repetitive DNA sequences^15^ was used to process the SGRP reads against the relevant consensus sequence, thereby identifying rDNA-specific shotgun reads. However, on manual examination some reads that passed through this filtering process were suspected to be non-rDNA in origin. Performing a BLASTN search of selected reads, identified by TURNIP as variant but mapping to highly conserved rDNA regions, against the National Center for Biotechnology Information Nucleotide database^26^ resulted in identifying a possible contamination of the *S. paradoxus* reference strain CBS 432 with *Plasmodium falciparum* sequence. More stringent filtering strategies were therefore employed prior to further use with the TURNIP software.

### Data Filtering

A custom Perl script (Supplementary Figure S5), employing BioPerl modules [ref. 27, BioPerl version 1.6.9, Perl version 5.12], was used to filter the dataset more stringently. Sequencing reads from each strain were aligned to the *S. paradoxus* consensus sequence using blastall [ref. 28, BLAST version 2.2.27+, using BioPerl module Bio::Tools::Run::StandAloneBlast]. Conditions for reads to pass the filter comprised a minimum read length of 150 bp, minimum identity of 75%, and minimum percentage of the original read involved in a High-scoring Segment Pair of 75%. BLAST parameters included an E-value of 1 × 10^−10^, gap opening penalty of 3, gap extension penalty of 1 and a nucleotide mismatch penalty of −1. The results of this modified filter were compared to the previous results. All reads that were complicit in a putative polymorphism in the original filtering process, but not in the more stringent modified filter, and a random selection of remaining reads were then manually checked, confirming that the final results were highly likely to retain all true hits to the rDNA sequence while removing false positive matches. Reads that passed the new filter were then converted into a BLAST searchable database using formatdb, ready for use with TURNIP. This process resulted in a total of 36,522 and 44,481 rDNA-specific sequencing reads for the 26 *S. paradoxus* and 34 *S. cerevisiae* strains respectively.

### Variant Discovery and Analysis

Three categories of inter-genomic polymorphism - SNPs, insertions and deletions - and their respective intra-genomic polymorphism forms - pSNPs, pINSs and pDELs, were identified within the two strain datasets using TURNIP (see the Results section for definitions of all polymorphism types). Default parameters were used within the configuration file, with a minimum quality score of 38, and an allowed shortness of 38. The TURNIP BLAST parameters -b and -v were each set to 800, higher than the default value, to allow all reads aligning to specific rDNA regions to be stored and analysed. To ensure confidence in unresolved variation discovery, the additional criterion that pSNPs, pINSs and pDELs should be present in more than a single read in a given strain was asserted. The output was then inspected visually for complex mutations (denoted CX), i.e. nucleotide positions at which more than one type of variant was identified^11^. These positions were then annotated manually. Strain- and species-specific variants were analysed within the R statistical computing environment [ref. 29, version 2.15.2], using standard and MASS libraries (version 7.3-22) together with the fitdistrplus library [ref. 30, version 1.0-2] for fitting of Beta distributions to allele frequency curves.

## Additional Information

**How to cite this article**: James, S. A. *et al*. Prevalence and Dynamics of Ribosomal DNA Micro-heterogeneity Are Linked to Population History in Two Contrasting Yeast Species. *Sci. Rep.* **6**, 28555; doi: 10.1038/srep28555 (2016).

## Supplementary Material

Supplementary Information

## Figures and Tables

**Table 1:** rDNA Sequence Variation Uncovered Within the *S. paradoxus* Dataset.

Strain	Population	Inter-genomic variants			Intra-genomic variants			CX	Total
		SNP	INS	DEL	pSNP	pINS	pDEL		
Q89.8	European	0	1	0	0	0	0	0	1
Q32.3	European	0	1	0	0	0	1	0	2
S36.7	European	0	1	1	0	0	0	0	2
Q95.3	European	0	1	1	0	1	0	0	3
Y7.2	European	1	1	0	0	0	1	0	3
Z1.1	European	1	1	1	0	0	0	0	3
T21.4	European	0	1	0	0	1	1	1	4
Y6.5	European	1	1	0	0	1	2	0	5
Q62.5	European	2	1	0	2	0	1	0	6
Q59.1	European	0	1	0	5	2	0	1	9
CBS 432 (T)	European	5	2	1	0	0	2	0	10
DBVPG 4650	European	2	1	0	4	1	3	0	11
CBS 5829	European	6	2	1	3	0	0	0	12
KPN 3828	European	7	2	3	1	0	0	0	13
KPN 3829	European	7	2	2	1	0	1	0	13
N-17	European	1	2	0	16	2	6	1	28
IFO 1804	Far Eastern	39	2	4	0	1	0	0	46
N-44	Far Eastern	38	3	3	1	0	1	0	46
N-43	Far Eastern	40	4	5	1	0	0	0	50
N-45	Far Eastern	4	1	0	36	4	5	0	50
A12	American	84	9	10	0	0	0	0	103
A4	American	88	10	6	0	0	0	1	105
UFRJ 50816	American	91	6	10	0	1	0	0	108
YPS138	American	95	8	7	0	0	0	0	110
UFRJ 50791	American	95	10	8	0	0	0	0	113
DBVPG 6304	American	97	6	6	2	1	1	1	114
Total		704	80	69	72	15	25	5	970

Table of variation for each *S. paradoxus* strain, compared to the reference strain CBS 432, as identified using the TURNIP software. The three fixed, inter-genomic variants - single nucleotide polymorphism, insertion and deletion - are denoted as SNP, INS and DEL respectively. Their unresolved, intra-genomic forms - partial single nucleotide polymorphism, partial insertion and partial deletion - are denoted as pSNP, pINS and pDEL respectively. Complex mutations, the manifestation of different mutation types at a single rDNA site, are denoted as CX. For each strain, the population from which they derive is also given. Ordering the strains by total polymorphism count results in the strains being split into their population groups.

**Table 2:** rDNA Sequence Variation Uncovered Within the *S. cerevisiae* Dataset.

Strain	Genome Type	Group	Inter-genomic variants			Intra-genomic variants			CX	Total
			SNP	INS	DEL	pSNP	pINS	pDEL		
W303	Mosaic	OM	0	0	0	3	0	1	0	4
L_1374	Structured mosaic	W/E	6	0	7	2	0	1	0	16
DBVPG 1106	Structured mosaic	W/E	7	0	9	1	0	1	0	18
DBVPG 1788	Structured mosaic	W/E	8	0	11	0	0	0	0	19
YJM975	Structured mosaic	W/E	6	0	7	4	0	3	0	20
YJM978	Structured mosaic	W/E	6	0	7	4	0	3	0	20
YJM981	Structured mosaic	W/E	6	0	6	4	0	5	0	21
YPS128	Structured clean	NA	14	0	10	0	0	0	0	24
S288c	Mosaic	OM	0	0	0	14	1	10	0	25
BC187	Structured mosaic	W/E	7	0	5	7	1	5	0	25
DBVPG 1373	Structured mosaic	W/E	8	0	5	7	1	4	0	25
YPS606	Structured clean	NA	14	0	9	2	0	2	0	27
NCYC 110	Structured clean	WA+	15	2	8	2	0	1	0	28
DBVPG 6765	Structured mosaic	W/E	13	0	8	3	0	4	0	28
DBVPG 6044	Structured clean	WA+	15	1	9	2	1	1	0	29
SK1	Mosaic	WA+	16	0	7	3	0	3	0	29
UWOPS87-2421	Mosaic	UM	14	0	11	4	0	1	0	30
322134S	Mosaic	OM	6	0	5	12	2	5	0	30
Y9	Structured mosaic	SA	8	1	6	10	3	4	1	33
Y55	Mosaic	WA+	15	1	9	7	0	2	0	34
273614N	Mosaic	OM	4	2	6	15	1	6	0	34
Y12	Structured mosaic	SA	9	1	6	11	4	5	0	36
378604X	Mosaic	OM	0	0	1	20	4	11	0	36
DBVPG 6040	Mosaic	OM	0	0	0	27	2	11	0	40
K11	Structured mosaic	SA	23	5	9	2	0	2	0	41
YS9	Mosaic	OM	1	0	1	27	4	8	0	41
YIIc17_E5	Mosaic	YII	7	0	6	18	4	6	0	41
UWOPS05-227-2	Structured clean	MA	24	0	11	7	0	0	0	42
UWOPS05-217-3	Structured clean	MA	27	0	7	3	0	6	0	43
UWOPS83-787-3	Mosaic	UM	8	0	6	21	1	7	0	43
UWOPS03-461-4	Structured clean	MA	29	0	15	0	0	0	0	44
NCYC 361	Mosaic	OM	0	0	0	27	4	13	0	44
YS4	Mosaic	OM	9	1	6	24	4	5	0	49
DBVPG 1853	Mosaic	OM	14	1	2	18	5	9	0	49
Total			339	15	215	311	42	145	1	1068

Table of variation for each *S. cerevisiae* strain, compared to the reference strain S288c, as identified using the TURNIP software. The seven mutation types - single nucleotide polymorphism, insertion, deletion, partial single nucleotide polymorphism, partial insertion, partial deletion and complex mutation - are again denoted as SNP, INS, DEL, pSNP, pINS, pDEL and CX respectively. For each strain, the genome type (mosaic, structured clean or structured mosaic) and the geographic/industrial group (MA [Malaysian], NA [North American], SA [Sake], WA+ [West African + other mosaics], W/E [Wine/European], YII [strain YIIc17-E5], UM [UWOPS mosaics] or OM [Other Mosaics]), as determined in^14^, are also given.

**Table 3:** Estimates of Type 1 and Type 2 partial insertions and deletions in *S. paradoxus* and *S. cerevisiae*.

Species	Group	pINS			pDEL		
		Type 1	Type 2	Total	Type 1	Type 2	Total
*S. paradoxus*	European	8	0	8	16	2	18
	Far Eastern	2	3	5	5	1	6
	American	2	0	2	0	1	1
*S. cerevisiae*	Structured clean	1	0	1	4	6	10
	Structured mosaic	5	4	9	16	21	37
	Mosaic	22	10	32	66	32	98

*S. paradoxus* strains are subdivided by their population groups and *S. cerevisiae* strains by their genome structure groups.

**Table 4:** Region-By-Region Breakdown of rDNA Variation in *S. paradoxus* and *S. cerevisiae*.

Region	*S. paradoxus*								*S. cerevisiae*							
	Inter-genomic variants			Intra-genomic variants			CX	Total	Inter-genomic variants			Intra-genomic variants			CX	Total
	SNP	INS	DEL	pSNP	pINS	pDEL			SNP	INS	DEL	pSNP	pINS	pDEL		
26S	7	0	0	1	0	0	0	8	4	0	0	18	2	1	0	25
ETS2	77	25	26	7	1	7	1	144	4	0	9	12	0	12	0	37
IGS1	237	48	37	30	14	15	4	385	110	11	125	122	20	97	1	486
5S	0	0	0	0	0	1	0	1	0	0	0	0	0	0	0	0
IGS2	316	7	6	19	0	0	0	348	178	4	11	111	9	10	0	323
ETS1	48	0	0	9	0	1	0	58	24	0	54	18	0	16	0	112
18S	2	0	0	0	0	0	0	2	0	0	0	9	0	1	0	10
ITS1	15	0	0	5	0	0	0	20	19	0	0	18	5	0	0	42
5.8S	0	0	0	0	0	0	0	0	0	0	0	0	0	0	0	0
ITS2	2	0	0	1	0	1	0	4	0	0	16	3	6	8	0	33
Total	704	80	69	72	15	25	5	970	339	15	215	311	42	145	1	1068

The number of polymorphisms of each type split according to different regions of the ribosomal DNA unit for *S. paradoxus* and *S. cerevisiae*.

## References

[b1] Álvarez I. & Wendel J. F. Ribosomal ITS sequences and plant phylogenetic inference. Mol Phylogenet Evol 29, 417–34 (2003). 14615184 10.1016/s1055-7903(03)00208-2

[b2] Buckler E. S., Ippolito A. & Holtsford T. P. The evolution of ribosomal DNA: divergent paralogues and phylogenetic implications. Genetics 145, 821–32 (1997). 9055091 10.1093/genetics/145.3.821 PMC1207866

[b3] Kiss L. Limits of nuclear ribosomal DNA internal transcribed spacer (ITS) sequences as species barcodes for Fungi. Proc Natl Acad Sci USA 109, E1811; author reply E1812 (2012). 22715287 10.1073/pnas.1207143109 PMC3390822

[b4] Nilsson R. H., Kristiansson E., Ryberg M., Hallenberg N. & Larsson K.-H. Intraspecific ITS variability in the kingdom fungi as expressed in the international sequence databases and its implications for molecular species identification. Evol Bioinform Online 4, 193–201 (2008). 19204817 10.4137/ebo.s653 PMC2614188

[b5] Ganley A. R. & Kobayashi T. Highly efficient concerted evolution in the ribosomal DNA repeats: total rDNA repeat variation revealed by whole-genome shotgun sequence data. Genome Res 17, 184–91 (2007). 17200233 10.1101/gr.5457707 PMC1781350

[b6] Vĕtrovský T. & Baldrian P. The Variability of the 16S rRNA Gene in Bacterial Genomes and Its Consequences for Bacterial Community Analyses. PLoS ONE 8, e57923 (2013). 23460914 10.1371/journal.pone.0057923 PMC3583900

[b7] Yip W., Vincent N. G. & Baserga S. J. Ribonucleoproteins in Archaeal Pre-rRNA Processing and Modification. Archaea 614735 (2013). 23554567 10.1155/2013/614735 PMC3608112

[b8] Petes T. D. Yeast ribosomal DNA genes are located on chromosome XII. Proc Natl Acad Sci USA 76, 410–414 (1979). 370829 10.1073/pnas.76.1.410 PMC382949

[b9] Eickbush T. H. & Eickbush D. G. Finely orchestrated movements: evolution of the ribosomal RNA genes. Genetics 175, 477–85 (2007). 17322354 10.1534/genetics.107.071399 PMC1800602

[b10] Simon U. K. & Weiß M. Intragenomic variation of fungal ribosomal genes is higher than previously thought. Mol Biol Evol 25, 2251–4 (2008). 18728073 10.1093/molbev/msn188

[b11] James S. A. . Repetitive sequence variation and dynamics in the ribosomal DNA array of Saccharomyces cerevisiae as revealed by whole-genome resequencing. Genome Res 19, 626–35 (2009). 19141593 10.1101/gr.084517.108 PMC2665781

[b12] The Saccharomyces Genome Resequencing Project. Available at: http://www.sanger.ac.uk/research/projects/genomeinformatics/sgrp.html (Date of access: 15th January 2016).

[b13] Liti G. . Population genomics of domestic and wild yeasts. Nature 458, 337–41 (2009). 19212322 10.1038/nature07743 PMC2659681

[b14] West C., James S. A., Davey R. P., Dicks J. & Roberts I. N. Ribosomal DNA Sequence Heterogeneity Reflects Intraspecies Phylogenies and Predicts Genome Structure in Two Contrasting Yeast Species. Syst Biol 63, 543–554 (2014). 24682414 10.1093/sysbio/syu019 PMC4055870

[b15] Davey R. P., James S. A., Dicks J. & Roberts I. N. TURNIP: tracking unresolved nucleotide polymorphisms in large hard-to-assemble regions of repetitive DNA sequence. Bioinformatics 26, 2908–9 (2010). 20926422 10.1093/bioinformatics/btq557

[b16] Strand M., Prolla T. A., Liskay R. M. & Petes T. D. Destabilization of tracts of simple repetitive DNA in yeast by mutations affecting DNA mismatch repair. Nature 365, 274–6 (1993). 8371783 10.1038/365274a0

[b17] Loftus B. J. . The genome of the basidiomycetous yeast and human pathogen Cryptococcus neoformans. Science 307, 1321–4 (2005). 15653466 10.1126/science.1103773 PMC3520129

[b18] Chakraborty R., Fuerst P. A. & Nei M. Statistical studies on protein polymorphism in natural populations. III. Distribution of allele frequencies and the number of alleles per locus. Genetics 94, 1039–63 (1980). 17249018 10.1093/genetics/94.4.1039 PMC1214178

[b19] Haegeman B. & Weitz J. S. A neutral theory of genome evolution and the frequency distribution of genes. BMC Genomics 13, 196 (2012). 22613814 10.1186/1471-2164-13-196 PMC3386021

[b20] McStay B. & Grummt I. The Epigenetics of rRNA Genes: From Molecular to Chromosome Biology. Annual Review of Cell and Developmental Biology 24, 131–157 (2008). 10.1146/annurev.cellbio.24.110707.175259 18616426

[b21] Relethford J. H. Human Population Genetics (John Wiley & Sons, Inc., Hoboken, New Jersey, 2012).

[b22] Ganley A. R. D. & Kobayashi T. Monitoring the Rate and Dynamics of Concerted Evolution in the Ribosomal DNA Repeats of *Saccharomyces cerevisiae* Using Experimental Evolution. Mol Biol Evol 28, 2883–2891 (2013). 21546356 10.1093/molbev/msr117

[b23] van Sluis M. & McStay B. Ribosome biogenesis: Achilles heel of cancer? Genes Cancer 5, 152–153 (2014). 25061498 10.18632/genesandcancer.14 PMC4104764

[b24] Benson D. A. . GenBank. Nucleic Acids Res 41, D36–D42 (2013). 23193287 10.1093/nar/gks1195 PMC3531190

[b25] The Saccharomyces Genome Database. Available at: http://www.yeastgenome.org (Date of access: 15th January 2016).

[b26] National Center for Biotechnology Information. Available at: http://www.ncbi.nlm.nih.gov (Date of access: 15th January 2016).

[b27] Stajich J. E. . The Bioperl toolkit: Perl modules for the life sciences. Genome Res 12, 1611–8 (2002). 12368254 10.1101/gr.361602 PMC187536

[b28] Altschul S. F., Gish W., Miller W., Myers E. W. & Lipman D. J. Basic local alignment search tool. J Mol Biol 215, 403–410 (1990). 2231712 10.1016/S0022-2836(05)80360-2

[b29] R. Core Team. *R: A Language and Environment for Statistical Computing.* R Foundation for Statistical Computing, Vienna, Austria. URL http://www.R-project.org. (Date of access: 15th January 2016) (2013).

[b30] Delignette-Muller M. L., Pouillot R., Denis J. B. & Dutang C. *Package ‘fitdistrplus’. Help to Fit of a Parametric Distribution to Non-Censored or Censored data*. URL https://cran.r-project.org/web/packages/fitdistrplus/index.html. (Date of access: 15th January 2016) (2012).

