# Supplementary material for: Prevalence and Dynamics of Ribosomal DNA Micro-heterogeneity Are Linked to Population History in Two Contrasting Yeast Species
Source: Sci Rep. 2016 Jun 27;6:28555. doi: 10.1038/srep28555 (PMC4921842; doi:10.1038/srep28555)
Supplement: Supplementary Information [file srep28555-s1.pdf]

Supplementary Figures and Tables for:

Prevalence and Dynamics of Ribosomal DNA Micro-heterogeneity Are Linked to Population History in Two Contrasting Yeast Species

Stephen A. James[1,+], Claire West[1,3,+], Robert P. Davey[2], Jo Dicks[1,*], and Ian N. Roberts[1,*]

[1]National Collection of Yeast Cultures, Institute of Food Research, Norwich Research Park, Norwich, NR4 7UA, UK
[2]Bioinformatics, The Genome Analysis Centre, Norwich Research Park, Norwich, NR4 7UH, UK
[3]Current address: Department of Crop Genetics, John Innes Centre, Norwich Research Park, Norwich, NR4 7UH, UK

[*]E-mail:ian.roberts@ifr.ac.uk or jo.dicks@ifr.ac.uk
[+]these authors contributed equally to this work

Supplementary Figure S1: examples of partial insertions, deletions and complex variants

a) A Type 1 partial deletion in *S. cerevisiae*

Deletion variant identified in the *S. cerevisiae* IGS1 region (TURNIP alignment positions 4101 to 4116) of strain 378604X

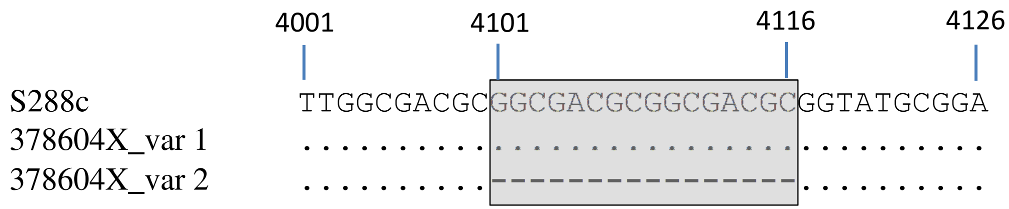

Sequence Type	Tract length	Frequency
378604X_var 1[A]	16	28.6% (14 reads)
378604X_var 2[B]	0	71.4% (35 reads)

[A] Identical in length to S288c (reference strain); [B] Dominant sequence variant

In *S. cerevisiae* strain 378604X, this 16 nucleotide tract has been deleted from approximately 70% of its rDNA units. Almost all of the strains are found to possess deletions in this area (see Supplementary Table S2). However, most are fixed deletions.

b) A Type 2 partial insertion in *S. paradoxus*

Variable length homopolymeric polyT tract found in the *S. paradoxus* IGS1 region (TURNIP alignment positions 3929 to 3937) of strain N-45

```
               3919      3929                              3939
CBS 432        GGGGACATAATTTTTTTTT------------------------AAGTAATGGC
N-45_var 1     ...................------------------------..........
N-45_var 2     ...................TTTTTTTTTTTTTTTTTTTTTTT-..........
N-45_var 3     ...................TTTTTTTTTTTTTTTTTTTTTTTT..........
```

Sequence Type	Tract length	Frequency
N-45_var 1[A]	9	6.1% (2 reads)
N-45_var 2	32	9.1% (3 reads)
N-45_var 3[B]	33	84.8% (28 reads)

[A] Identical in length to CBS 432 (reference strain); [B] Dominant sequence variant

In the reference strain, this homopolymeric tract comprises of 9 T residues. However, in both IFO 1804 and N-45 it appears to be variable in length. As we see above, in N-45 three different variants were detected, one identical in length to the reference strain, and two significantly longer (32 T's and 33 T's), with the longest length variant (33 T's) found in the majority of covering reads (28/33). In contrast, although significantly longer than the reference strain, this tract appears to be of fixed length in the other two Far Eastern strains (N-43, 23 T's; N-44, 26 T's).

c) Homopolymeric tracts

Location and size of the five largest IGS1 poly(dA).(dT) tracts in *S. cerevisiae* (S288c) and their equivalent counterparts in *S. paradoxus* (CBS 432)

**Tract type:**	**Location**		**Length**	
	**S288c**	**CBS 432**	**S288c**	**CBS 432**
polyT	3627-3642	3638-3653	16	16
polyA	3834-3841	3856-3861	8	6
polyT	3914-3935	3930-3938	22	9
polyT	4300-4316	Absent	17	0
polyA	4487-4515	4479-4495	29	17

In the *S. cerevisiae* reference strain S288c, these five tracts range from 8 to 29 residues in length. Some of these tract-specific deletions are fixed while others are partial variants. For example, in S288c we observe a 16 residue poly(dT) tract between base positions 3627 and 3642. In the soil strain DBVPG 1788, this same tract is shorter and only 13 residues in length, whereas in the beer spoilage strain NCYC 361, it exists in two variant forms, one identical in length to S288c (16 T residues) and present on the majority of covering reads, and a shorter variant (12 T residues) present on only six covering reads. These results indicate that not only can homopolymeric tracts vary in length between different rDNA arrays of the same species, but they can also vary in length between individual repeats of the same rDNA array (i.e. a Type 2 partial variant). In contrast in the *S. paradoxus* reference strain (CBS 432^{T}), there are only four equivalent poly(dA).poly(dT) tracts in the IGS1 region, and two of these are significantly shorter in length than their *S. cerevisiae* counterparts.

d) <u>A complex mutation in *S. paradoxus*</u>

A SNP/DEL complex mutation at positions 4049 to 4050 was putatively identified in the European *S. paradoxus* strains T21.4 and Q59.1. A number of reads derived from each of the strains exhibit a 'C' nucleotide instead of a 'T' at position 4050 (adjacent to an 'A' at position 4049), with the remaining reads showing a deletion of positions 4049 and 4050. Crucially, a T→C SNP was identified at position 4050 of the closely related strain Y6.5. We therefore hypothesise that such a SNP was also harboured by T21.4 and Q59.1 (i.e. the polymorphism became fixed in an ancestor of T21.4, Q59.1 and Y6.5), with the deletion event occurring subsequently.

```
                4039      4049       4060
                 |         |          |
CBS 432         ACACACACACATATATATATAT
T21.4_var 1     ..........AC..........
T21.4_var 2     ..........--..........
```

Sequence Type	Frequency
T21.4_var 1[A]	34.9% (21 reads 4049 /23 reads 4050)
T21.4_var 2[B]	65.1% (41 reads)

[A] Identical in length to CBS 432 (reference strain); [B] Dominant sequence variant

Supplementary Figure S2: Unit occupancy distributions

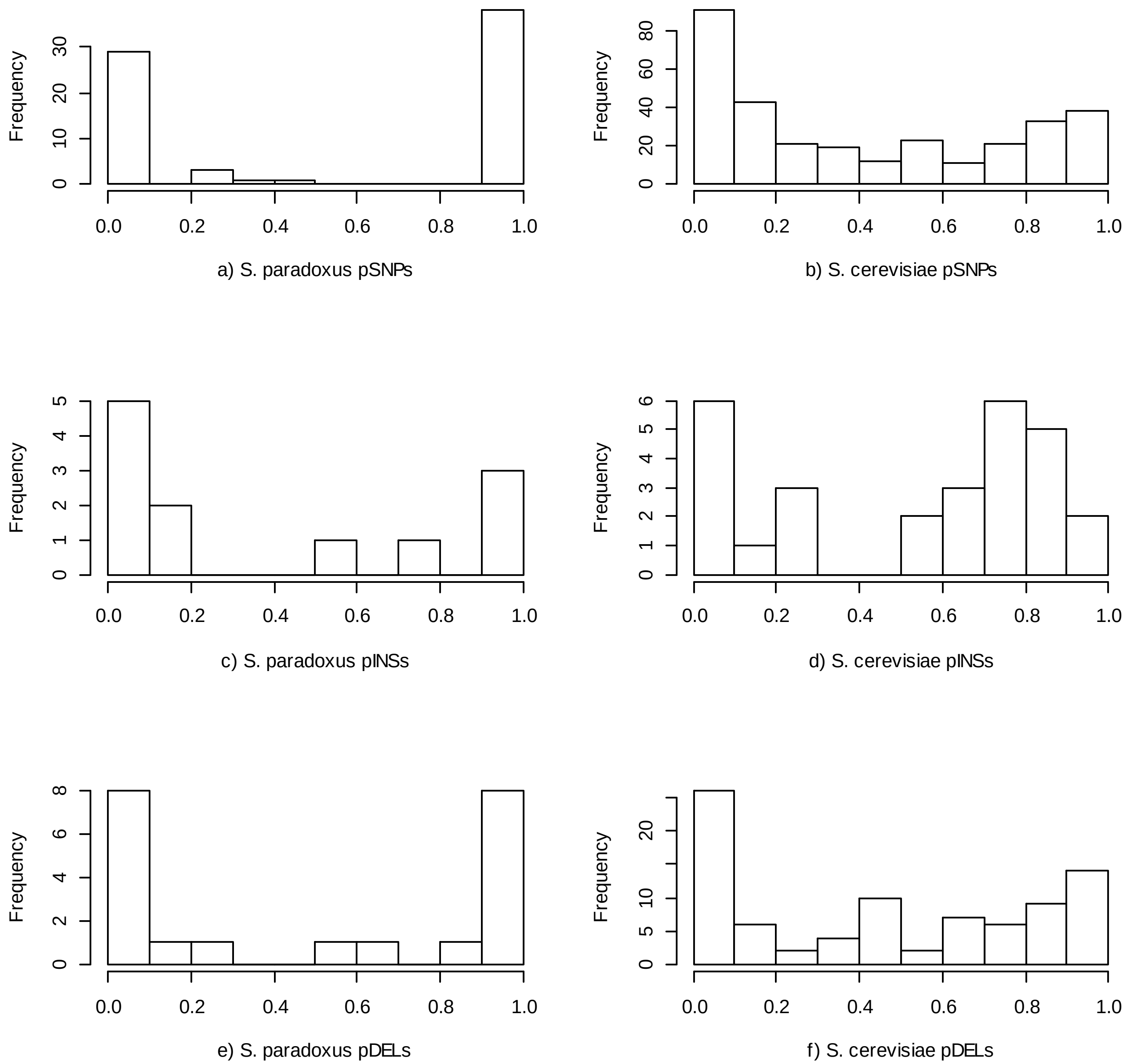

Supplementary Figure S3: Differing pSNP occupancy distributions between Structured clean, Structured mosaic and Mosaic strains of *Saccharomyces cerevisiae*

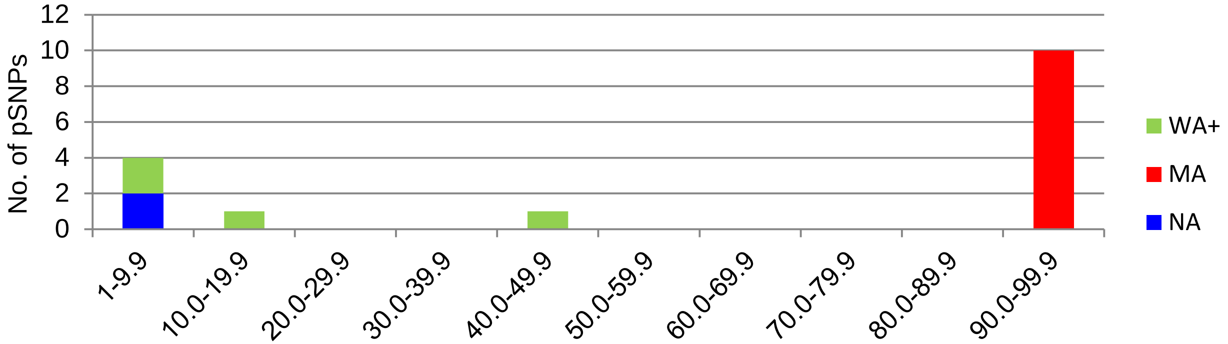

a) Percentage occupancies of 16 rDNA pSNPs in 7 Structured clean *Saccharomyces cerevisiae* strains

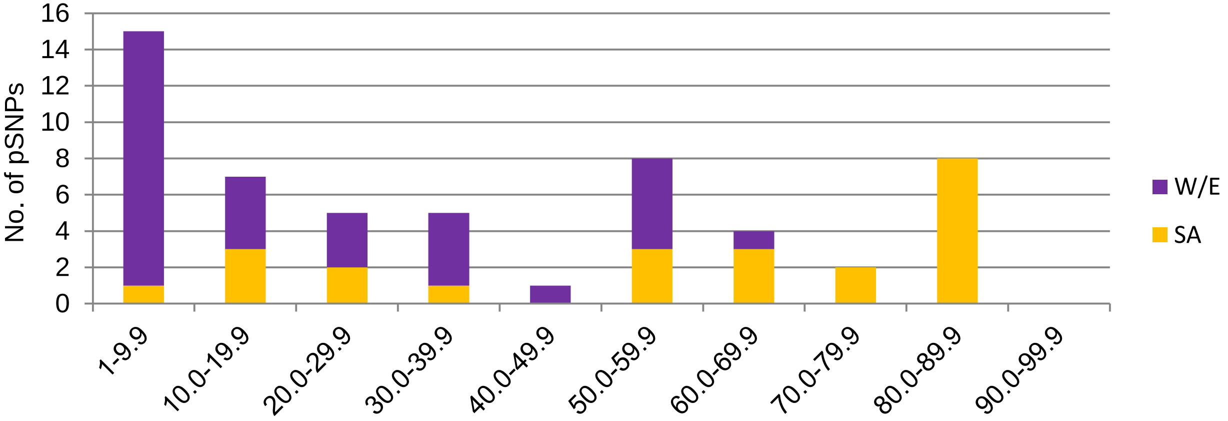

b) Percentage occupancies of 55 rDNA pSNPs in 12 Structured mosaic *Saccharomyces cerevisiae* strains

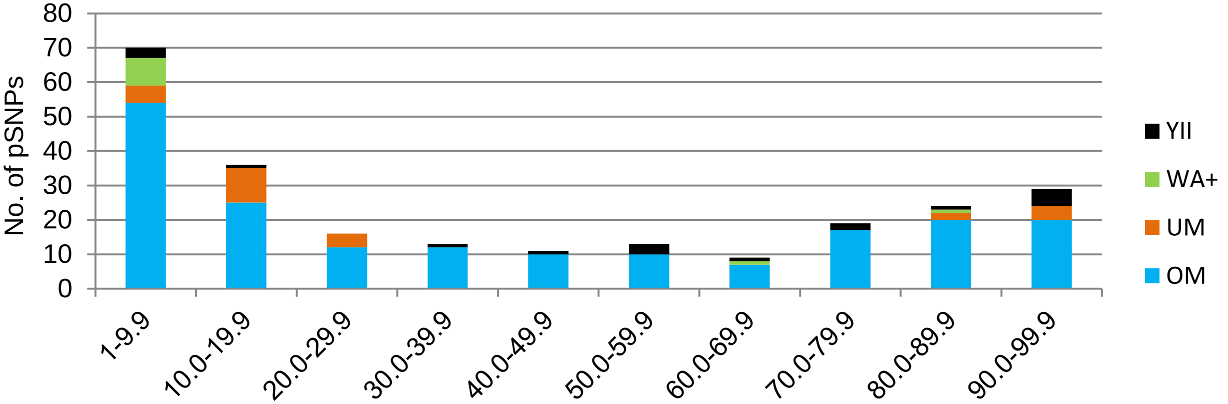

c) Percentage occupancies of 240 rDNA pSNPs in 15 Mosaic *Saccharomyces cerevisiae* strains

Supplementary Figure S4: Sequence read coverage

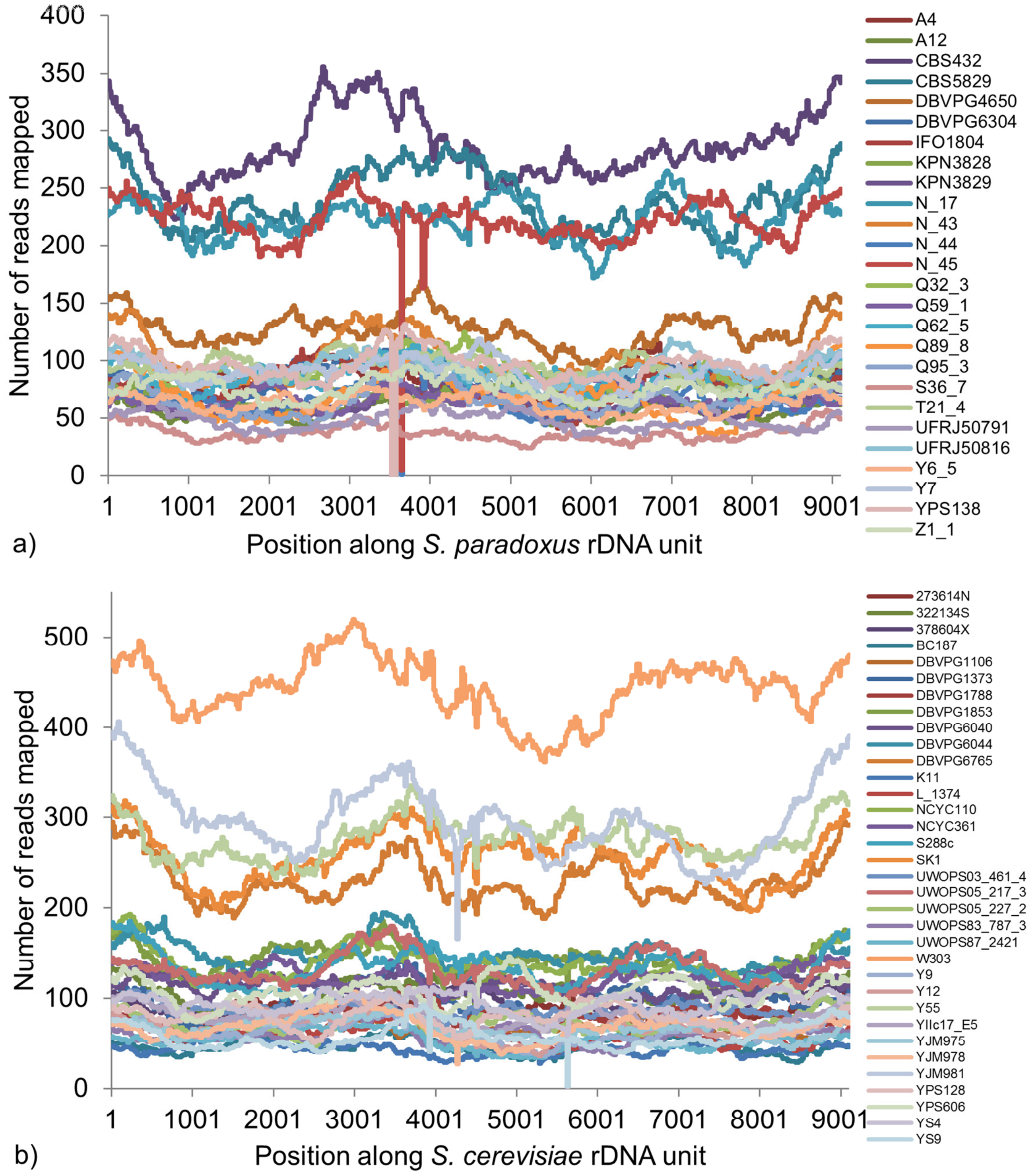

For the 26 *Saccharomyces paradoxus* strains, most coverage values were found to range from 40 to 100, though with four strains (CBS432, CBS5829, N-17 and N-45) ranging from 180 to 360. All six American strains and two Far Eastern strains (N-44 and N-45) were found to possess small (up to 20 bp) sections within the ETS2/IGS1 region that were either not covered or very poorly covered. The ETS2 and IGS1 regions have been shown to display high quantities of polymorphisms in *S. paradoxus* (see main text Fig. 3 and Table 4). When the 20 bp windows flanking these areas of poor/no coverage were examined in detail, it was discovered that all eight strains had SNPs, insertions and/or deletions on either side of these coverage anomalies. This implied that any reads spanning these areas were either too dissimilar to the reference consensus to pass the BLAST or multiple alignment filters within the read mapping procedure, or else carried large deletions. For example, all six American strains have consistently no coverage over one specific area (ETS2 region, positions 3520 to 3540, and 3560 to 3580), which would appear to be a feature of this group and its diversification from the species type strain, which derives from Europe. For the 34 *Saccharomyces cerevisiae* strains, the read coverage of most strains fell within the range 40 to 200, although five strains (W303, Y55, YJM981, SK1 and DBVPG6765) were found to possess a sequence read coverage of between 200 and 480. Two strains (the closely related YS4 and YS9) both exhibited only two or three mapped reads in a single small area, positions 5620-5639.

Supplementary Figure S5

```
#!/usr/bin/perl -w

#script to try blast against consensus sequences to remove contamination and reads from other areas of the genome
#v2, added scoring HSPs, only allowing those above min identity and % of original read length.
#v3 changed subs to be called from one, filter_strains. Added sub to remove all of the blast outfiles (as too many and too much space taken up),
#plus added sub to run formatdb on the filtered fasta output.
#used in James SA, West C, Davey RP, Dicks J and Roberts IN. Prevalence and Dynamics of Ribosomal DNA Micro-heterogeneity Are Linked to Population History in Two Contrasting Yeast Species.

use strict;
use warnings;
use Bio::SearchIO;
use Bio::Seq;
use Bio::SeqIO;
use Bio::Tools::Run::StandAloneBlast;
use Data::Dumper;
use File::Path;
use Cwd;
use File::Copy;


#variables
my $db_dir = "../database_files/";#location of blast database for consensus
my $strain_dir = "../strains/";#location of strains
my $db_name = "wrapped_Sc_consensus_sequence.fa";#consensus strain to compare to
my $strain_name;
my $strain_suffix = ".fasta";#file name suffix of strains
my $results_dir;
my @reads= ();

#specify names of strains to filter
my @strains =("YJM789");#"273614N","322134S","378604X","BC187","DBVPG1106","DBVPG1373","DBVPG1788","DBVPG1853","DBVPG6040","DBVPG6044","DBVPG6765","K11","L_1374","L_1528","NCYC110","NCYC361","S288c","SK1","UWOPS03_461_4","UWOPS05_217_3","UWOPS05_227_2","UWOPS83_787_3","UWOPS87_2421","W303","Y12","Y55","Y9","YIIc17_E5","YJM789","YJM975","YJM978","YJM981","YPS128","YPS606","YS2","YS4","YS9");
#"A12","A4","CBS432","CBS5829","DBVPG4650","DBVPG6304","IFO1804","KPN3828","KPN3829","N_17","N_43","N_44","N_45","Q32_3","Q59_1","Q62_5","Q89_8","Q95_3","S36_7","T21_4","UFRJ50791","UFRJ50816","UWOPS91_917_1","Y6_5","Y7","YPS138","Z1_1"
my $min_read_len = 150;#min length of read
my $min_identity = 75;#min %identity of hit
my $min_percent = 75;#minimum % of original read involved


&filter_strains;

#run for all of the strains in the array
sub filter_strains
{
	foreach my $strain_name (@strains)
	{
		if(-e $strain_dir.$strain_name.$strain_suffix)#if the file exists
```

```
		{
			my $results_dir = "../results/$strain_name";

			if(!(-e $results_dir))
			{
			mkdir "../results/$strain_name";
			}

			&get_reads($strain_name);#read in sequence for that strain
			&blast_reads($results_dir);#blast against the consensus
			&rm_blast_dir($strain_name);#clear blast dir
			&format_fasta($strain_name);#make new filtered file into a blastdb
			@reads= ();#clear reads array
		}
	}

}

#read in sequence file for a strain
sub get_reads
{
	my $the_strain = shift;
	my $seqio_obj = Bio::SeqIO->new(-file => $strain_dir.$the_strain.$strain_suffix, -format => "fasta" );

	while (my $seq_obj = $seqio_obj->next_seq)
	{
		print $seq_obj->id, "\n";

		#if read length is greater than minimum, store it
		if(length($seq_obj->seq)>=$min_read_len)
		{
			#store reads in an array
			push(@reads, $seq_obj);
		}
		else
		{
			print "Read".$seq_obj->id." not stored!!"
		}
	}

	my $rlen = @reads;
	print "There are $rlen reads in this strain\n";
}

#BLAST parameters and call blast (used in "blast_reads" sub)
sub get_blast {
   my $results_dir = shift;
   my $read_id = shift;
   my $input = shift;
   my $db = shift;
   my $cores = 2;

   #Inputs dna sequence string (any FASTA should work) and outputs record containing all BLAST alignments
   my $blast_outfile = "$results_dir/$read_id-blast.out";
   my @bparams = ('program' => 'blastn', 'database' => $db, 'outfile' => $blast_outfile);
   my $factory = Bio::Tools::Run::StandAloneBlast->new(@bparams);
```

```
    #custom params
    $factory->X(1); #x dropoff
    $factory->G(3); #gap open pen
    $factory->E(1); #gap extend pen
    $factory->q(-1); #nucleotide mismatch pen
    $factory->g("T"); #gapped alignment
    $factory->e(1e-10); #evalue
    $factory->F("F"); #filters
    $factory->a($cores); #CPUs/cores

    my $blast_report = $factory->blastall($input);
    if (!-e $blast_outfile) { die "Cannot open $blast_outfile! Cannot continue...\n"; }
    else {
       my $blastObj = new Bio::SearchIO(-format => 'blast', -file => $blast_outfile);
       return $blastObj;
    }
}


# blast each read from the strain using "get_blast" sub, writing to file all those which pass threshold of
#%id and length
sub blast_reads
{
        my $results_dir = shift;

        my $good = 0;

        #file to write to
        my $seqio_obj2 = Bio::SeqIO->new(-file => '>filtered.fasta', -format => 'fasta' );

        #for each read, blast
        for(my $i=0; $i<(@reads);$i++)
        {
                my $input = $reads[$i];
                my $read_id = $input->id;
                my $read_orig_len = length($input->seq);
                my $pass = 0;

                my $bl = get_blast($results_dir,$read_id, $input, $db_dir.$db_name);

        my $result = $bl->next_result();
        my @alignments = $result->hits;

        print "$i blast done\n";

        foreach my $alignment (@alignments)
        {
                my $hsp;
                while( my $h = $alignment->next_hsp())
                {
                if ($h->rank == 1) { $hsp = $h; }
                last;
                }

                if (defined $hsp)
```

```
			{
			#if hsp has > min percent identity and is > min size of read involved, set read to pass
			if ($hsp->percent_identity >= $min_identity && $hsp->length('query') >= (($read_orig_len/100)*
$min_percent) )
				{
					$pass = 1;
				}
			 }
		}#end of foreach

		if($pass==1)#if the read passes thresholds for %id and min size, write that seq to file
		{
			$good++;
			$seqio_obj2->write_seq($reads[$i]);
		}
		}

		#all reads done
		print "$good reads passed\n";
}

#remove blast dir for the strain
sub rm_blast_dir
{
		my $strain_name = shift;
		my $dir = "../results/$strain_name";
		rmtree([$dir]);
		print "Blast results deleted for $strain_name \n";

}

#run formatdb on the fasta file
sub format_fasta
{
		my $strain_name = shift;
		#get the current working directory
		 my $pwd = cwd();
		# print "$pwd - formatdb files to go in here\n";

		 my $output_dir = "../output/$strain_name";

		 if(!(-e $output_dir))
			{
			mkdir "../output/$strain_name";
			print "made output directory\n";
			}

		#copy output file into correct dir
		my $oldfile = "filtered.fasta";
		my $newlocation = "../output/$strain_name/$strain_name"."_filtered$strain_suffix";
		move($oldfile, $newlocation);

		 #run formatdb
		 system("formatdb", "-i","$newlocation","-o","T", "-p","F");

}
```

Supplementary Table S1. *Saccharomyces paradoxus* and *Saccharomyces cerevisiae* strains

***S. paradoxus* strain**	**Source**	**Geographic location**	**Population**
A4	Bark of *Quercus rubra*	Mont St-Hilaire, Quebec, Canada	American
A12	Soil beneath *Q. rubra*	Mont St-Hilaire, Quebec, Canada	American
CBS 432[A,NT]	Bark of *Quercus* sp.	Moscow area, Russia	European
CBS 5829	Mor soil (pH 3.6)	Denmark	European
DBVPG 4650	Fossilized guano in a cavern	Marche, Italy	European
DBVPG 6304	*Drosophila pseudoobscura*	Yosemite, California, USA	American
IFO 1804	Bark of *Quercus* sp.	Japan	Far Eastern
KPN 3828	Bark of *Q. rubra*	Novosibirsk, Siberia, Russia	European
KPN 3829	Bark of *Q. rubra*	Novosibirsk, Siberia, Russia	European
N-17	Exudate of *Q. robur*	Tatarstan, Russia	European
N-43	Exudate of *Q. mongolica*	Vladivostok, Russia	Far Eastern
N-44	Exudate of *Q. mongolica*	Terney, Russia	Far Eastern
N-45	Exudate of *Q. mongolica*	Terney, Russia	Far Eastern
Q32.3	Bark of *Quercus* sp.	Windsor Great Park, UK	European
Q59.1	Bark of *Quercus* sp.	Windsor Great Park, UK	European
Q62.5	Bark of *Quercus* sp.	Windsor Great Park, UK	European
Q89.8	Bark of *Quercus* sp.	Windsor Great Park, UK	European
Q95.3	Bark of *Quercus* sp.	Windsor Great Park, UK	European
S36.7	Bark of *Quercus* sp.	Silwood Park, UK	European
T21.4	Bark of *Quercus* sp.	Silwood Park, UK	European
UFRJ 50791	*Drosophila* sp.	Catalao Point, Rio de Janeiro, Brazil	American
UFRJ 50816	*Drosophila* sp.	Tijuca Forest, Rio de Janeiro, Brazil	American
Y6.5	Bark of *Quercus* sp.	Silwood Park, UK	European
Y7.2	Bark of *Quercus* sp.	Silwood Park, UK	European
YPS138	Soil beneath *Q. velutina*	Pennsylvania, USA	American
Z1.1	Bark of *Quercus* sp.	Silwood Park, UK	European

[A]Reference strain; [NT]Neotype strain

S. cerevisiae strain	Source	Geographic location	Genome type[C]	Lineage[C]
27361N	Clinical isolate (fecal)	Royal Victoria Infirmary, Newcastle, UK	Mosaic	OM
322134S	Clinical isolate (Throat-sputum)	Royal Victoria Infirmary, Newcastle, UK	Mosaic	OM
378604X	Clinical isolate (Sputum)	Royal Victoria Infirmary, Newcastle, UK	Mosaic	OM
BC187	Barrel fermentation	Napa Valley, USA	Structured mosaic	W/E
DBVPG 1106	Grapes	Australia	Structured mosaic	W/E
DBVPG 1373	Soil	Netherlands	Structured mosaic	W/E
DBVPG 1788	Soil	Turku, Finland	Structured mosaic	W/E
DBVPG 1853	White Teff	Ethiopia	Mosaic	OM
DBVPG 6040	Fermenting fruit juice	Netherlands	Mosaic	OM
DBVPG 6044	Bili wine, from *Osbeckia grandiflora*	West Africa	Structured clean	WA+
DBVPG 6765	Unknown	Unknown	Structured mosaic	W/E
K11	Shochu sake strain	Japan	Structured mosaic	SA
L_1374	Fermentation from must Pais	Cauquenes, Chile	Structured mosaic	W/E
NCYC 110	Ginger beer from *Z. officinale*	West Africa	Structured clean	WA+
NCYC 361	Beer spoilage strain from wort	Ireland	Mosaic	OM
S288c[A,B]	Rotting fig	Merced, California, USA	Mosaic	OM
SK1[B]	Soil	USA	Mosaic	WA+
UWOPS03-461-4	Nectar, Bertram palm	Telok Senangin, Malaysia	Structured clean	MA
UWOPS05-217-3	Nectar, Bertram palm	Telok Senangin, Malaysia	Structured clean	MA
UWOPS05-227-2	Stingless bee (*Trigona* sp.)	Telok Senangin, Malaysia	Structured clean	MA
UWOPS83-787-3	Fruit, *Opuntia stricta*	Great Inagua Island, Bahamas	Mosaic	UM
UWOPS87-2421	Cladode, *Opuntia megacantha*	Puhelu Road, Maui, Hawaii	Mosaic	UM
W303[B]	Laboratory generated	NA	Mosaic	OM
Y12	Palm wine strain	Ivory Coast	Structured mosaic	SA
Y55[B]	Grape	France	Mosaic	WA+
Y9	Ragi (similar to sake wine)	Indonesia	Structured mosaic	SA
YIIc17_E5	Wine	Sauternes, France	Mosaic	YII
YJM975	Vaginal isolate from patient with vaginitis	Ospedali Riuniti di Bergamo, Italy	Structured mosaic	W/E
YJM978	Vaginal isolate from patient with vaginitis	Ospedali Riuniti di Bergamo, Italy	Structured mosaic	W/E
YJM981	Vaginal isolate from patient with vaginitis	Ospedali Riuniti di Bergamo, Italy	Structured mosaic	W/E
YPS128	Soil beneath *Quercus alba*	Pennsylvania, USA	Structured clean	NA
YPS606	Bark of *Q. rubra*	Pennsylvania, USA	Structured clean	NA
YS4	Baker's strain	Netherlands	Mosaic	OM
YS9	Baker's strain	Singapore	Mosaic	OM

[A]Reference strain; [B]Laboratory strain

[C]Classification according to West *et al.* (2014) [13]: MA [Malaysian], NA [North American], SA [Sake], W/E [Wine/European], WA+ [West African + other mosaics], YII [strain YIIc17-E5], UM [UWOPS mosaics] or OM [Other Mosaics]

Supplementary Table S2: SNPs and pSNPs in *Saccharomyces paradoxus* strains

Position	Region	No. of strains	SNPs	pSNPs	Strains										
248	26S	6	6	0	A12	A4	UFRJ50816	YPS138	UFRJ50791	DBVPG6304					
543	26S	1	1	0	N-43										
1174	26S	1	0	1	N-45										
3456	ETS2	5	3	2	N-17	IFO1804	N-44	N-43	N-45						
3517	ETS2	2	2	0	UFRJ50816	DBVPG6304									
3547	ETS2	6	6	0	A12	A4	UFRJ50816	YPS138	UFRJ50791	DBVPG6304					
3548	ETS2	5	5	0	Q62.5	IFO1804	N-44	N-43	N-45						
3549	ETS2	4	4	0	IFO1804	N-44	N-43	N-45							
3550	ETS2	4	4	0	A4	YPS138	UFRJ50791	DBVPG6304							
3551	ETS2	4	3	1	IFO1804	N-44	N-43	N-45							
3553	ETS2	2	2	0	A12	UFRJ50816									
3554	ETS2	4	4	0	A4	YPS138	UFRJ50791	DBVPG6304							
3555	ETS2	4	4	0	A4	YPS138	UFRJ50791	DBVPG6304							
3556	ETS2	4	4	0	A4	YPS138	UFRJ50791	DBVPG6304							
3557	ETS2	4	4	0	A4	YPS138	UFRJ50791	DBVPG6304							
3558	ETS2	8	6	2	Q62.5	Q59.1	A12	A4	UFRJ50816	YPS138	UFRJ50791	DBVPG6304			
3559	ETS2	8	6	2	Q62.5	Q59.1	A12	A4	UFRJ50816	YPS138	UFRJ50791	DBVPG6304			
3586	ETS2	6	6	0	A12	A4	UFRJ50816	YPS138	UFRJ50791	DBVPG6304					
3587	ETS2	6	6	0	A12	A4	UFRJ50816	YPS138	UFRJ50791	DBVPG6304					
3595	ETS2	5	5	0	A12	A4	UFRJ50816	UFRJ50791	DBVPG6304						
3599	ETS2	1	1	0	YPS138										
3601	ETS2	1	1	0	YPS138										
3603	ETS2	1	1	0	YPS138										
3619	IGS1	6	6	0	A12	A4	UFRJ50816	YPS138	UFRJ50791	DBVPG6304					
3631	IGS1	1	0	1	N-17										
3635	IGS1	3	3	0	UFRJ50816	YPS138	DBVPG6304								
3637	IGS1	2	2	0	A12	DBVPG6304									
3638	IGS1	1	1	0	DBVPG6304										
3643	IGS1	3	3	0	A12	A4	YPS138								
3645	IGS1	1	0	1	DBVPG6304										
3667	IGS1	6	6	0	A12	A4	UFRJ50816	YPS138	UFRJ50791	DBVPG6304					
3668	IGS1	2	2	0	UFRJ50816	UFRJ50791									
3691	IGS1	6	6	0	A12	A4	UFRJ50816	YPS138	UFRJ50791	DBVPG6304					
3728	IGS1	5	3	2	N-17	IFO1804	N-44	N-43	N-45						
3746	IGS1	5	3	2	N-17	IFO1804	N-44	N-43	N-45						
3747	IGS1	5	3	2	N-17	IFO1804	N-44	N-43	N-45						
3759	IGS1	6	6	0	A12	A4	UFRJ50816	YPS138	UFRJ50791	DBVPG6304					
3760	IGS1	6	6	0	A12	A4	UFRJ50816	YPS138	UFRJ50791	DBVPG6304					
3769	IGS1	2	2	0	UFRJ50816	UFRJ50791									
3775	IGS1	11	9	2	N-17	IFO1804	N-44	N-43	N-45	A12	A4	UFRJ50816	YPS138	UFRJ50791	DBVPG6304
3818	IGS1	5	3	2	N-17	IFO1804	N-44	N-43	N-45						
3821	IGS1	6	6	0	A12	A4	UFRJ50816	YPS138	UFRJ50791	DBVPG6304					
3824	IGS1	6	6	0	A12	A4	UFRJ50816	YPS138	UFRJ50791	DBVPG6304					
3842	IGS1	5	3	2	N-17	IFO1804	N-44	N-43	N-45						
3925	IGS1	1	1	0	DBVPG6304										
3926	IGS1	1	1	0	DBVPG6304										
3927	IGS1	1	1	0	DBVPG6304										
3944	IGS1	1	1	0	Y7.2										
3960	IGS1	6	6	0	A12	A4	UFRJ50816	YPS138	UFRJ50791	DBVPG6304					
3967	IGS1	6	6	0	A12	A4	UFRJ50816	YPS138	UFRJ50791	DBVPG6304					
3995	IGS1	6	6	0	A12	A4	UFRJ50816	YPS138	UFRJ50791	DBVPG6304					
4014	IGS1	3	3	0	UFRJ50816	UFRJ50791	DBVPG6304								
4024	IGS1	6	6	0	A12	A4	UFRJ50816	YPS138	UFRJ50791	DBVPG6304					
4028	IGS1	1	0	1	N-17										
4041	IGS1	1	0	1	N-43										
4046	IGS1	3	3	0	A4	YPS138	DBVPG6304								
4048	IGS1	6	6	0	A12	A4	UFRJ50816	YPS138	UFRJ50791	DBVPG6304					

4050	IGS1	8	7	1	Y6.5	DBVPG4650	CBS5829	KPN3828	KPN3829	IFO1804	N-44	N-43									
4052	IGS1	4	3	1	CBS5829	IFO1804	N-44	N-43													
4054	IGS1	4	3	1	CBS5829	IFO1804	N-43	N-45													
4056	IGS1	1	0	1	CBS5829																
4065	IGS1	1	1	0	YPS138																
4066	IGS1	1	1	0	YPS138																
4067	IGS1	10	8	2	CBS432	DBVPG4650	CBS5829	KPN3828	KPN3829	IFO1804	N-44	N-43	YPS138	DBVPG6304							
4068	IGS1	9	8	1	CBS432	DBVPG4650	CBS5829	KPN3828	KPN3829	IFO1804	N-44	N-43	YPS138								
4250	IGS1	2	2	0	UFRJ50816	UFRJ50791															
4288	IGS1	6	6	0	A12	A4	UFRJ50816	YPS138	UFRJ50791	DBVPG6304											
4289	IGS1	6	6	0	A12	A4	UFRJ50816	YPS138	UFRJ50791	DBVPG6304											
4295	IGS1	6	6	0	A12	A4	UFRJ50816	YPS138	UFRJ50791	DBVPG6304											
4296	IGS1	17	15	2	Q59.1	CBS432	DBVPG4650	CBS5829	KPN3828	KPN3829	N-17	IFO1804	N-44	N-43	N-45	A12	A4	UFRJ50816	YPS138	UFRJ50791	DBVPG6304
4323	IGS1	5	5	0	A12	A4	YPS138	UFRJ50791	DBVPG6304												
4378	IGS1	4	3	1	IFO1804	N-44	N-43	N-45													
4379	IGS1	4	3	1	IFO1804	N-44	N-43	N-45													
4380	IGS1	4	3	1	IFO1804	N-44	N-43	N-45													
4385	IGS1	4	3	1	IFO1804	N-44	N-43	N-45													
4395	IGS1	2	2	0	UFRJ50816	UFRJ50791															
4408	IGS1	6	6	0	A12	A4	UFRJ50816	YPS138	UFRJ50791	DBVPG6304											
4418	IGS1	6	6	0	A12	A4	UFRJ50816	YPS138	UFRJ50791	DBVPG6304											
4454	IGS1	10	9	1	IFO1804	N-44	N-43	N-45	A12	A4	UFRJ50816	YPS138	UFRJ50791	DBVPG6304							
4465	IGS1	6	6	0	A12	A4	UFRJ50816	YPS138	UFRJ50791	DBVPG6304											
4470	IGS1	6	6	0	A12	A4	UFRJ50816	YPS138	UFRJ50791	DBVPG6304											
4647	IGS2	1	1	0	DBVPG6304																
4655	IGS2	10	9	1	IFO1804	N-44	N-43	N-45	A12	A4	UFRJ50816	YPS138	UFRJ50791	DBVPG6304							
4659	IGS2	6	6	0	A12	A4	UFRJ50816	YPS138	UFRJ50791	DBVPG6304											
4679	IGS2	4	3	1	IFO1804	N-44	N-43	N-45													
4684	IGS2	10	9	1	IFO1804	N-44	N-43	N-45	A12	A4	UFRJ50816	YPS138	UFRJ50791	DBVPG6304							
4687	IGS2	6	6	0	A12	A4	UFRJ50816	YPS138	UFRJ50791	DBVPG6304											
4724	IGS2	6	6	0	A12	A4	UFRJ50816	YPS138	UFRJ50791	DBVPG6304											
4733	IGS2	6	6	0	A12	A4	UFRJ50816	YPS138	UFRJ50791	DBVPG6304											
4735	IGS2	4	3	1	IFO1804	N-44	N-43	N-45													
4748	IGS2	2	2	0	UFRJ50816	UFRJ50791															
4773	IGS2	6	6	0	A12	A4	UFRJ50816	YPS138	UFRJ50791	DBVPG6304											
4792	IGS2	4	3	1	IFO1804	N-44	N-43	N-45													
4814	IGS2	6	6	0	A12	A4	UFRJ50816	YPS138	UFRJ50791	DBVPG6304											
4840	IGS2	6	6	0	A12	A4	UFRJ50816	YPS138	UFRJ50791	DBVPG6304											
4859	IGS2	6	6	0	A12	A4	UFRJ50816	YPS138	UFRJ50791	DBVPG6304											
4879	IGS2	10	9	1	IFO1804	N-44	N-43	N-45	A12	A4	UFRJ50816	YPS138	UFRJ50791	DBVPG6304							
4943	IGS2	2	2	0	UFRJ50816	UFRJ50791															
4973	IGS2	6	6	0	A12	A4	UFRJ50816	YPS138	UFRJ50791	DBVPG6304											
4977	IGS2	2	2	0	A12	A4															
5003	IGS2	6	6	0	A12	A4	UFRJ50816	YPS138	UFRJ50791	DBVPG6304											
5011	IGS2	1	0	1	KPN3828																
5015	IGS2	6	6	0	A12	A4	UFRJ50816	YPS138	UFRJ50791	DBVPG6304											
5029	IGS2	6	6	0	A12	A4	UFRJ50816	YPS138	UFRJ50791	DBVPG6304											
5030	IGS2	6	6	0	A12	A4	UFRJ50816	YPS138	UFRJ50791	DBVPG6304											
5057	IGS2	6	6	0	A12	A4	UFRJ50816	YPS138	UFRJ50791	DBVPG6304											
5059	IGS2	6	6	0	A12	A4	UFRJ50816	YPS138	UFRJ50791	DBVPG6304											
5072	IGS2	6	6	0	A12	A4	UFRJ50816	YPS138	UFRJ50791	DBVPG6304											
5116	IGS2	1	1	0	DBVPG6304																
5126	IGS2	10	9	1	IFO1804	N-44	N-43	N-45	A12	A4	UFRJ50816	YPS138	UFRJ50791	DBVPG6304							
5182	IGS2	6	6	0	A12	A4	UFRJ50816	YPS138	UFRJ50791	DBVPG6304											
5225	IGS2	6	6	0	A12	A4	UFRJ50816	YPS138	UFRJ50791	DBVPG6304											
5325	IGS2	10	9	1	IFO1804	N-44	N-43	N-45	A12	A4	UFRJ50816	YPS138	UFRJ50791	DBVPG6304							
5326	IGS2	5	5	0	CBS432	DBVPG4650	CBS5829	KPN3828	KPN3829												
5331	IGS2	6	6	0	A12	A4	UFRJ50816	YPS138	UFRJ50791	DBVPG6304											
5334	IGS2	4	3	1	IFO1804	N-44	N-43	N-45													
5338	IGS2	6	6	0	A12	A4	UFRJ50816	YPS138	UFRJ50791	DBVPG6304											
5343	IGS2	1	1	0	YPS138																

5347	IGS2	11	9	2	N-17	IFO1804	N-44	N-43	N-45	A12	A4	UFRJ50816	YPS138	UFRJ50791	DBVPG6304
5356	IGS2	1	1	0	DBVPG6304										
5375	IGS2	6	6	0	A12	A4	UFRJ50816	YPS138	UFRJ50791	DBVPG6304					
5402	IGS2	6	6	0	A12	A4	UFRJ50816	YPS138	UFRJ50791	DBVPG6304					
5426	IGS2	6	6	0	A12	A4	UFRJ50816	YPS138	UFRJ50791	DBVPG6304					
5435	IGS2	4	3	1	IFO1804	N-44	N-43	N-45							
5445	IGS2	6	6	0	A12	A4	UFRJ50816	YPS138	UFRJ50791	DBVPG6304					
5484	IGS2	6	6	0	A12	A4	UFRJ50816	YPS138	UFRJ50791	DBVPG6304					
5487	IGS2	6	6	0	A12	A4	UFRJ50816	YPS138	UFRJ50791	DBVPG6304					
5548	IGS2	6	6	0	A12	A4	UFRJ50816	YPS138	UFRJ50791	DBVPG6304					
5577	IGS2	6	6	0	A12	A4	UFRJ50816	YPS138	UFRJ50791	DBVPG6304					
5634	IGS2	4	3	1	IFO1804	N-44	N-43	N-45							
5640	IGS2	6	6	0	A12	A4	UFRJ50816	YPS138	UFRJ50791	DBVPG6304					
5642	IGS2	3	2	1	IFO1804	N-45	UFRJ50791								
5644	IGS2	3	2	1	N-44	N-43	N-45								
5645	IGS2	6	6	0	A12	A4	UFRJ50816	YPS138	UFRJ50791	DBVPG6304					
5655	IGS2	5	5	0	CBS432	DBVPG4650	CBS5829	KPN3828	KPN3829						
5667	IGS2	2	2	0	UFRJ50816	UFRJ50791									
5669	IGS2	6	6	0	A12	A4	UFRJ50816	YPS138	UFRJ50791	DBVPG6304					
5700	IGS2	6	6	0	A12	A4	UFRJ50816	YPS138	UFRJ50791	DBVPG6304					
5715	IGS2	6	6	0	A12	A4	UFRJ50816	YPS138	UFRJ50791	DBVPG6304					
5767	IGS2	6	6	0	A12	A4	UFRJ50816	YPS138	UFRJ50791	DBVPG6304					
5792	IGS2	1	1	0	DBVPG6304										
5804	IGS2	2	2	0	KPN3828	KPN3829									
5817	IGS2	1	0	1	N-45										
5825	IGS2	7	6	1	N-45	A12	A4	UFRJ50816	YPS138	UFRJ50791	DBVPG6304				
5846	IGS2	6	5	1	IFO1804	N-44	N-43	N-45	A12	A4					
5879	ETS1	6	6	0	A12	A4	UFRJ50816	YPS138	UFRJ50791	DBVPG6304					
6045	ETS1	3	0	3	Q59.1	N-17	N-45								
6104	ETS1	1	0	1	Q59.1										
6116	ETS1	2	2	0	Z1.1	Q62.5									
6296	ETS1	11	9	2	N-17	IFO1804	N-44	N-43	N-45	A12	A4	UFRJ50816	YPS138	UFRJ50791	DBVPG6304
6321	ETS1	4	3	1	IFO1804	N-44	N-43	N-45							
6415	ETS1	1	1	0	YPS138										
6418	ETS1	6	6	0	A12	A4	UFRJ50816	YPS138	UFRJ50791	DBVPG6304					
6436	ETS1	1	0	1	KPN3829										
6494	ETS1	6	6	0	A12	A4	UFRJ50816	YPS138	UFRJ50791	DBVPG6304					
6510	ETS1	10	9	1	IFO1804	N-44	N-43	N-45	A12	A4	UFRJ50816	YPS138	UFRJ50791	DBVPG6304	
6514	ETS1	6	6	0	A12	A4	UFRJ50816	YPS138	UFRJ50791	DBVPG6304					
6742	18S	2	2	0	UFRJ50816	UFRJ50791									
8377	ITS1	1	0	1	N-44										
8395	ITS1	6	6	0	A12	A4	UFRJ50816	YPS138	UFRJ50791	DBVPG6304					
8509	ITS1	6	6	0	A12	A4	UFRJ50816	YPS138	UFRJ50791	DBVPG6304					
8528	ITS1	1	0	1	N-17										
8532	ITS1	5	3	2	N-17	IFO1804	N-44	N-43	N-45						
8619	ITS1	1	0	1	N-17										
8951	ITS2	1	0	1	N-17										
9098	ITS2	2	2	0	UFRJ50816	UFRJ50791									
		**776**	**704**	**72**											

Supplementary Table S2: pSNP frequencies in *Saccharomyces paradoxus* strains

Position	Region	pSNPs	Strains		Reference	Variants	Frequencies
1174	26S	1	N-45		192	2	0.010
3456	ETS2	2	N-17	N-45	182	3	0.016
					4	124	0.969
3551	ETS2	1	N-45		4	92	0.958
3558	ETS2	2	Q62.5	Q59.1	55	2	0.035
					34	2	0.056
3559	ETS2	2	Q62.5	Q59.1	55	2	0.035
					34	2	0.056
3631	IGS1	1	N-17		182	3	0.016
3645	IGS1	1	DBVPG6304		3	50	0.943
3728	IGS1	2	N-17	N-45	188	2	0.011
					4	85	0.955
3746	IGS1	2	N-17	N-45	183	2	0.011
					3	96	0.970
3747	IGS1	2	N-17	N-45	184	2	0.011
					3	96	0.970
3775	IGS1	2	N-17	N-45	180	2	0.011
					4	51	0.927
3818	IGS1	2	N-17	N-45	181	4	0.022
					3	78	0.963
3842	IGS1	2	N-17	N-45	183	4	0.021
					3	119	0.975
4028	IGS1	1	N-17		148	5	0.033
4041	IGS1	1	N-43		60	2	0.032
4050	IGS1	1	DBVPG4650		67	36	0.350
4052	IGS1	1	CBS5829		5	207	0.976
4054	IGS1	1	CBS5829		5	206	0.976
4056	IGS1	1	CBS5829		7	205	0.967
4067	IGS1	2	DBVPG4650	DBVPG6304	47	19	0.288
					64	2	0.030

4068	IGS1	1	DBVPG4650			47	19	0.288
4296	IGS1	2	Q59.1	DBVPG4650		48	2	0.040
						70	29	0.293
4378	IGS1	1	N-45			6	107	0.947
4379	IGS1	1	N-45			6	92	0.939
4380	IGS1	1	N-45			6	91	0.938
4385	IGS1	1	N-45			6	99	0.943
4454	IGS1	1	N-45			4	122	0.968
4655	IGS2	1	N-45			5	146	0.967
4679	IGS2	1	N-45			6	140	0.959
4684	IGS2	1	N-45			7	138	0.952
4735	IGS2	1	N-45			6	140	0.959
4792	IGS2	1	N-45			5	154	0.969
4879	IGS2	1	N-45			3	148	0.980
5011	IGS2	1	KPN3828			63	3	0.045
5126	IGS2	1	N-45			3	122	0.976
5325	IGS2	1	N-45			3	122	0.976
5334	IGS2	1	N-45			3	132	0.978
5347	IGS2	2	N-17	N-45		179	2	0.011
						4	124	0.969
5435	IGS2	1	N-45			4	124	0.969
5634	IGS2	1	N-45			2	97	0.980
5642	IGS2	1	N-45			2	92	0.979
5644	IGS2	1	N-45			3	92	0.968
5817	IGS2	1	N-45			147	3	0.020
5825	IGS2	1	N-45			14	128	0.901
5846	IGS2	1	N-45			2	136	0.986
6045	ETS1	3	Q59.1	N-17	N-45	56	3	0.051
						2	136	0.986
						175	2	0.011
6104	ETS1	1	Q59.1			31	24	0.436
6296	ETS1	2	N-17	N-45		162	2	0.012
						2	121	0.984

6321	ETS1	1	N-45		2	128	0.985
6436	ETS1	1	KPN3829		43	2	0.044
6510	ETS1	1	N-45		2	131	0.985
8377	ITS1	1	N-44		46	3	0.061
8528	ITS1	1	N-17		191	2	0.010
8532	ITS1	2	N-17	N-45	189	2	0.010
					4	125	0.969
8619	ITS1	1	N-17		206	2	0.010
8951	ITS2	1	N-17		187	3	0.016
		**72**					

Supplementary Table S2: INSs and pINSs in *Saccharomyces paradoxus* strains

Position	Region	No. of strains	INSs	pINSs	Type 1 pINSs	Type 2 pINSs	Strains										
3515	ETS2	1	1	0	0	0	UFRJ50791										
3517	ETS2	3	3	0	0	0	A12	A4	YPS138								
3518	ETS2	1	1	0	0	0	DBVPG6304										
3519	ETS2	2	2	0	0	0	A12	UFRJ50816									
3547	ETS2	11	11	0	0	0	Q89.8	Q32.3	S36.7	Z1.1	T21.4	Q59.1	CBS432	DBVPG4650	CBS5829	KPN3828	KPN3829
3548	ETS2	3	3	0	0	0	Q95.3	Y7.2	Y6.5								
3549	ETS2	1	1	0	0	0	Q62.5										
3558	ETS2	4	3	1	0	1	IFO1804	N-44	N-43	N-45							
3626	IGS1	1	1	0	0	0	A4										
3627	IGS1	1	1	0	0	0	UFRJ50791										
3631	IGS1	2	2	0	0	0	A4	UFRJ50791									
3635	IGS1	3	3	0	0	0	A12	A4	UFRJ50791								
3636	IGS1	2	2	0	0	0	UFRJ50816	YPS138									
3637	IGS1	2	2	0	0	0	A4	UFRJ50791									
3638	IGS1	3	1	2	2	0	Q95.3	T21.4	A12								
3643	IGS1	1	1	0	0	0	UFRJ50791										
3645	IGS1	1	0	1	1	0	UFRJ50816										
3646	IGS1	1	0	1	1	0	Y6.5										
3647	IGS1	1	0	1	1	0	DBVPG6304										
3748	IGS1	4	3	1	1	0	CBS432	CBS5829	KPN3829	N-17							
3754	IGS1	2	1	1	1	0	DBVPG4650	KPN3828									
3761	IGS1	1	1	0	0	0	N-43										
3855	IGS1	3	3	0	0	0	A12	UFRJ50791	DBVPG6304								
3856	IGS1	2	2	0	0	0	A4	YPS138									
3861	IGS1	1	1	0	0	0	YPS138										
3910	IGS1	3	1	2	2	0	N-17	N-44	N-45								
3915	IGS1	1	1	0	0	0	N-43										
3925	IGS1	4	4	0	0	0	A12	A4	YPS138	UFRJ50791							
3927	IGS1	1	1	0	0	0	A4										
3929	IGS1	7	5	2	0	2	IFO1804	N-43	N-45	A12	UFRJ50816	UFRJ50791	DBVPG6304				
3938	IGS1	2	2	0	0	0	N-44	YPS138									
3969	IGS1	6	6	0	0	0	A12	A4	UFRJ50816	YPS138	UFRJ50791	DBVPG6304					
4011	IGS1	1	1	0	0	0	DBVPG6304										
4033	IGS1	1	0	1	1	0	Q59.1										
4041	IGS1	1	1	0	0	0	N-45										
4050	IGS1	1	1	0	0	0	N-17										
4052	IGS1	2	1	1	1	0	Q59.1	N-17									
4071	IGS1	1	0	1	1	0	N-45										
4252	IGS2	1	1	0	0	0	UFRJ50816										
5642	IGS2	5	5	0	0	0	A12	A4	UFRJ50816	YPS138	DBVPG6304						
5644	IGS2	1	1	0	0	0	IFO1804										
		**95**	**80**	**15**	**12**	**3**											

Supplementary Table S2: pINS frequencies in *Saccharomyces paradoxus* strains

Position	Region	Type 1 pINSs	Strains		Reference	Variants	Frequencies
3638	IGS1	2	Q95.3	T21.4	37	2	0.051
					57	12	0.174
3645	IGS1	1	UFRJ50816		10	40	0.800
3646	IGS1	1	Y6.5		14	15	0.517
3647	IGS1	1	DBVPG6304		4	50	0.926
3748	IGS1	1	N-17		181	5	0.027
3754	IGS1	1	DBVPG4650		85	19	0.183
3910	IGS1	2	N-17	N-45	184	2	0.011
					3	60	0.952
4033	IGS1	1	Q59.1		34	3	0.081
4052	IGS1	1	Q59.1		41	2	0.047
4071	IGS1	1	N-45		2	50	0.962
		**12**					

Supplementary Table S2: DELs and pDELs in *Saccharomyces paradoxus* strains

Start position	End position	Region	No. of strains	DELs	pDELs	Type 1 pDELs	Type 2 pDELs	Strains						
3516	3517	ETS2	7	4	3	3	0	CBS432	DBVPG4650	KPN3828	N-17	IFO1804	N-44	N-43
3519	3519	ETS2	1	1	0	0	0	KPN3829						
3521	3522	ETS2	2	1	1	1	0	N-43	N-45					
3540	3544	ETS2	6	6	0	0	0	A12	A4	UFRJ50816	YPS138	UFRJ50791	DBVPG6304	
3554	3554	ETS2	2	2	0	0	0	A12	UFRJ50816					
3554	3557	ETS2	1	0	1	1	0	N-17						
3557	3557	ETS2	2	2	0	0	0	A12	UFRJ50816					
3592	3596	ETS2	1	1	0	0	0	YPS138						
3596	3603	ETS2	6	5	1	1	0	N-44	N-43	N-45	A12	UFRJ50816	DBVPG6304	
3597	3603	ETS2	1	1	0	0	0	IFO1804						
3597	3604	ETS2	1	1	0	0	0	A4						
3598	3603	ETS2	1	1	0	0	0	UFRJ50791						
3598	3606	ETS2	1	0	1	1	0	N-17						
3604	3604	ETS2	1	1	0	0	0	YPS138						
3624	3625	IGS1	3	3	0	0	0	A12	UFRJ50816	DBVPG6304				
3624	3639	IGS1	2	1	1	1	0	N-44	N-45					
3624	3640	IGS1	1	1	0	0	0	N-43						
3624	3645	IGS1	1	1	0	0	0	IFO1804						
3627	3628	IGS1	1	1	0	0	0	YPS138						
3632	3641	IGS1	1	0	1	1	0	N-17						
3634	3634	IGS1	1	1	0	0	0	UFRJ50791						
3638	3641	IGS1	3	2	1	0	1	CBS432	KPN3828	KPN3829				
3640	3641	IGS1	1	1	0	0	0	CBS5829						
3751	3754	IGS1	6	6	0	0	0	A12	A4	UFRJ50816	YPS138	UFRJ50791	DBVPG6304	
4033	4034	IGS1	2	0	2	2	0	T21.4	Y6.5					
4043	4050	IGS1	1	1	0	0	0	CBS432						
4049	4050	IGS1	6	3	3	2	1	Q32.3	S36.7	Q95.3	Y7.2	Z1.1	Q62.5	
4061	4063	IGS1	1	0	1	0	1	DBVPG6304						
4063	4063	IGS1	4	4	0	0	0	A12	A4	UFRJ50816	UFRJ50791			
4068	4068	IGS1	3	3	0	0	0	A12	UFRJ50816	UFRJ50791				
4206	4215	IGS1	1	0	1	1	0	DBVPG4650						
4474	4480	IGS1	1	1	0	0	0	UFRJ50816						
4475	4480	IGS1	1	1	0	0	0	UFRJ50791						
4478	4478	IGS1	4	1	3	3	0	Y6.5	DBVPG4650	KPN3828	KPN3829			
4478	4480	IGS1	2	0	2	1	1	N-44	N-45					
4478	4481	IGS1	2	2	0	0	0	IFO1804	N-43					
4478	4482	IGS1	1	1	0	0	0	A4						
4480	4482	IGS1	2	2	0	0	0	A12	DBVPG6304					
4480	4483	IGS1	1	1	0	0	0	YPS138						
4539	4539	5S	1	0	1	1	0	N-17						
4710	4712	IGS2	6	6	0	0	0	A12	A4	UFRJ50816	YPS138	UFRJ50791	DBVPG6304	
6330	6330	ETS1	1	0	1	1	0	N-45						
8950	8950	ITS2	1	0	1	1	0	N-17						
			**94**	**69**	**25**	**21**	**4**							

Supplementary Table S2: pDEL frequencies in *Saccharomyces paradoxus* strains

Start position	End position	Region	Type 1 pDELs	Strains			Reference	Variants	Frequencies
3516	3517	ETS2	3	CBS432	DBVPG4650	N-17	3	169	0.983
							59	18	0.234
							143	6	0.040
3521	3522	ETS2	1	N-45			2	95	0.979
3554	3557	ETS2	1	N-17			116	3	0.025
3596	3603	ETS2	1	N-45			4	98	0.961
3598	3606	ETS2	1	N-17			189	4	0.021
3624	3639	IGS1	1	N-45			4	130	0.970
3632	3641	IGS1	1	N-17			180	3	0.016
4033	4034	IGS1	2	T21.4	Y6.5		20	31	0.608
							6	27	0.818
4049	4050	IGS1	2	Y7.2	Q62.5		3	66	0.957
							2	54	0.964
4206	4215	IGS1	1	DBVPG4650			37	51	0.580
4478	4478	IGS1	3	Y6.5	DBVPG4650	KPN3829	44	4	0.083
							59	13	0.181
							3	35	0.921
4478	4480	IGS1	1	N-45			3	96	0.970
4539	4539	5S	1	N-17			178	2	0.011
6330	6330	ETS1	1	N-45			136	8	0.056
8950	8950	ITS2	1	N-17			189	2	0.010
			**21**						

Supplementary Table S2: Complex variants in *Saccharomyces paradoxus* strains

Strain	Start position	End position	Region	Details
T21.4	4049	4050	IGS1	Likely a fixed SNP at 4050 t --> c (as for Y6.5) followed by a pDEL of 4049-4050
Q59.1	4049	4050	IGS1	Likely a fixed SNP at 4050 t --> c (as for Y6.5) followed by a pDEL of 4049-4050
N_17	3547	3547	ETS2	Likely a fixed insertion followed by a pSNP
A4	4068	4068	IGS1	Likely a fixed SNP at 4068 t --> a followed by a pDEL
DBVPG6304	4068	4068	IGS1	Likely a fixed SNP at 4068 t--> a followed by a pDEL

Supplementary Table S2: All variants in *Saccharomyces paradoxus* strains

Strain	SNPs	pSNPs	INSs	pINSs	DELs	pDELs	CX	Total
Q89.8	0	0	1	0	0	0	0	**1**
Q32.3	0	0	1	0	0	1	0	**2**
S36.7	0	0	1	0	1	0	0	**2**
Q95.3	0	0	1	1	1	0	0	**3**
Y7.2	1	0	1	0	0	1	0	**3**
Z1.1	1	0	1	0	1	0	0	**3**
T21.4	0	0	1	1	0	1	1	**4**
Y6.5	1	0	1	1	0	2	0	**5**
Q62.5	2	2	1	0	0	1	0	**6**
Q59.1	0	5	1	2	0	0	1	**9**
CBS432	5	0	2	0	1	2	0	**10**
DBVPG4650	2	4	1	1	0	3	0	**11**
CBS5829	6	3	2	0	1	0	0	**12**
KPN3828	7	1	2	0	3	0	0	**13**
KPN3829	7	1	2	0	2	1	0	**13**
N-17	1	16	2	2	0	6	1	**28**
IFO1804	39	0	2	1	4	0	0	**46**
N-44	38	1	3	0	3	1	0	**46**
N-43	40	1	4	0	5	0	0	**50**
N-45	4	36	1	4	0	5	0	**50**
A12	84	0	9	0	10	0	0	**103**
A4	88	0	10	0	6	0	1	**105**
UFRJ50816	91	0	6	1	10	0	0	**108**
YPS138	95	0	8	0	7	0	0	**110**
UFRJ50791	95	0	10	0	8	0	0	**113**
DBVPG6304	97	2	6	1	6	1	1	**114**
	**704**	**72**	**80**	**15**	**69**	**25**	**5**	**970**

Supplementary Table S2: SNPs and pSNPs in *Saccharomyces cerevisiae* strains

Position	Region	No. of strains	SNPs	pSNPs	Strains
253	26S	4	4	0	NCYC110 DBVPG6044 SK1 Y55
524	26S	2	0	2	DBVPG6040 NCYC361
679	26S	1	0	1	DBVPG6044
1112	26S	1	0	1	UWOPS87-2421
1426	26S	1	0	1	DBVPG1373
1813	26S	1	0	1	YS9
1852	26S	1	0	1	DBVPG6765
1887	26S	1	0	1	DBVPG1853
2017	26S	1	0	1	YJM981
2420	26S	1	0	1	DBVPG1373
2996	26S	1	0	1	YPS606
3012	26S	1	0	1	DBVPG6765
3096	26S	1	0	1	273614N
3154	26S	1	0	1	322134S
3177	26S	3	0	3	NCYC110 SK1 Y55
3266	26S	1	0	1	DBVPG1853
3407	ETS2	1	1	0	DBVPG1853
3430	ETS2	1	0	1	YS4
3494	ETS2	2	0	2	YS9 YS4
3517	ETS2	5	2	3	UWOPS87-2421 UWOPS05-227-2 UWOPS03-461-4 UWOPS05-217-3 UWOPS83-787-3
3538	ETS2	3	1	2	Y9 Y12 K11
3555	ETS2	2	0	2	Y9 Y12
3590	ETS2	1	0	1	NCYC361
3599	ETS2	1	0	1	NCYC361
3609	IGS1	1	0	1	Y55
3610	IGS1	1	0	1	Y55
3611	IGS1	1	0	1	Y55
3612	IGS1	2	0	2	Y55 YIIc17_E5
3624	IGS1	3	2	1	DBVPG1788 DBVPG6765 YIIc17_E5
3632	IGS1	1	1	0	SK1
3659	IGS1	28	16	12	DBVPG1106 YJM975 YJM978 YPS128 YJM981 NCYC110 YPS606 BC187 DBVPG6044 SK1 DBVPG1373 UWOPS87-2421 Y55 273614N Y9 Y12 UWOPS05-227-2 378604X K11 UWOPS03-461-4 DBVPG6040 UWOPS05-217-3 YS9 YIIc17_E5 UWOPS83-787-3 NCYC361 YS4 DBVPG1853
3678	IGS1	2	0	2	YS9 YS4
3687	IGS1	1	0	1	BC187
3697	IGS1	5	0	5	S288c Y9 Y12 DBVPG6040 YIIc17_E5
3713	IGS1	5	3	2	UWOPS87-2421 UWOPS05-227-2 UWOPS03-461-4 UWOPS05-217-3 UWOPS83-787-3
3724	IGS1	1	1	0	K11
3769	IGS1	2	1	1	YS9 YS4
3803	IGS1	2	2	0	YPS128 YPS606
3813	IGS1	15	10	5	L_1374 DBVPG1106 DBVPG1788 YJM975 YJM978 YJM981 BC187 DBVPG6765 DBVPG1373 322134S 273614N 378604X YIIc17_E5 NCYC361 DBVPG1853
3840	IGS1	2	1	1	S288c YIIc17_E5
3841	IGS1	1	1	0	YIIc17_E5
3871	IGS1	1	0	1	BC187
3902	IGS1	29	24	5	L_1374 DBVPG1106 DBVPG1788 YJM975 YJM978 YPS128 YJM981 S288c NCYC110 YPS606 BC187 DBVPG6765 DBVPG6044 SK1 DBVPG1373 UWOPS87-2421 322134S Y55 273614N UWOPS05-227-2 378604X UWOPS03-461-4 DBVPG6040 UWOPS05-217-3 YS9 YIIc17_E5 UWOPS83-787-3 NCYC361 YS4
3903	IGS1	2	0	2	YS9 YS4
3967	IGS1	1	0	1	Y55
3974	IGS1	7	1	6	UWOPS87-2421 322134S Y9 Y12 K11 UWOPS83-787-3 NCYC361
3989	IGS1	6	2	4	YPS128 YPS606 322134S DBVPG6040 YS9 YS4
4056	IGS1	1	0	1	YIIc17_E5
4058	IGS1	1	0	1	YIIc17_E5
4067	IGS1	2	2	0	YPS128 YPS606
4070	IGS1	1	0	1	YS4
4116	IGS1	2	2	0	UWOPS87-2421 DBVPG1853
4128	IGS1	1	1	0	DBVPG1853
4129	IGS1	2	1	1	YS4 DBVPG1853
4155	IGS1	1	1	0	DBVPG1853
4156	IGS1	6	0	6	322134S 273614N 378604X YS9 NCYC361 YS4
4165	IGS1	1	1	0	DBVPG1853
4166	IGS1	12	3	9	S288c 322134S 273614N Y9 Y12 378604X K11 DBVPG6040 YS9 YIIc17_E5 NCYC361 YS4
4183	IGS1	3	1	2	Y9 Y12 K11
4195	IGS1	4	4	0	NCYC110 DBVPG6044 SK1 Y55
4203	IGS1	5	2	3	322134S YS9 NCYC361 YS4 DBVPG1853
4215	IGS1	17	10	7	YPS128 S288c YPS606 UWOPS87-2421 322134S 273614N Y9 Y12 UWOPS05-227-2 378604X K11 UWOPS03-461-4 DBVPG6040 UWOPS05-217-3 YIIc17_E5 UWOPS83-787-3 NCYC361
4224	IGS1	2	0	2	YS9 YS4
4241	IGS1	1	0	1	YS4
4248	IGS1	7	2	5	322134S 273614N 378604X YS9 NCYC361 YS4 DBVPG1853
4270	IGS1	4	3	1	UWOPS05-227-2 UWOPS03-461-4 UWOPS05-217-3 UWOPS83-787-3
4284	IGS1	4	3	1	UWOPS05-227-2 UWOPS03-461-4 UWOPS05-217-3 UWOPS83-787-3
4305	IGS1	1	0	1	DBVPG6040
4307	IGS1	1	0	1	S288c
4310	IGS1	1	1	0	K11
4317	IGS1	1	1	0	K11
4334	IGS1	1	1	0	K11
4365	IGS1	4	3	1	UWOPS05-227-2 UWOPS03-461-4 UWOPS05-217-3 UWOPS83-787-3
4386	IGS1	4	3	1	UWOPS05-227-2 UWOPS03-461-4 UWOPS05-217-3 UWOPS83-787-3
4401	IGS1	1	0	1	DBVPG1853
4418	IGS1	1	0	1	Y12
4431	IGS1	1	0	1	W303
4484	IGS1	10	0	10	L_1374 YJM975 YJM981 BC187 SK1 Y12 K11 YS9 NCYC361 YS4
4495	IGS1	1	0	1	DBVPG1853
4496	IGS1	4	0	4	UWOPS05-217-3 YS9 YS4 DBVPG1853
4498	IGS1	3	0	3	273614N YS9 YS4
4499	IGS1	1	0	1	DBVPG1853
4508	IGS1	1	0	1	DBVPG1373
4652	IGS2	1	0	1	DBVPG6765
4657	IGS2	1	0	1	L_1374
4664	IGS2	2	0	2	322134S 378604X
4701	IGS2	4	1	3	UWOPS05-227-2 UWOPS03-461-4 UWOPS05-217-3 UWOPS83-787-3
4728	IGS2	1	0	1	DBVPG1853
4754	IGS2	3	0	3	S288c 378604X DBVPG6040
4763	IGS2	2	0	2	273614N 378604X
4769	IGS2	13	8	5	NCYC110 DBVPG6044 SK1 322134S Y55 UWOPS05-227-2 378604X UWOPS03-461-4 UWOPS05-217-3 YS9 UWOPS83-787-3 YS4 DBVPG1853
4786	IGS2	1	0	1	BC187
4809	IGS2	1	0	1	DBVPG6040
4817	IGS2	1	1	0	K11
4843	IGS2	4	4	0	NCYC110 DBVPG6044 SK1 Y55
4854	IGS2	7	3	4	UWOPS87-2421 UWOPS05-227-2 UWOPS03-461-4 DBVPG6040 UWOPS05-217-3 UWOPS83-787-3 NCYC361
4925	IGS2	4	2	2	UWOPS05-227-2 UWOPS03-461-4 UWOPS05-217-3 UWOPS83-787-3
4928	IGS2	4	2	2	UWOPS05-227-2 UWOPS03-461-4 UWOPS05-217-3 UWOPS83-787-3
4963	IGS2	1	0	1	DBVPG6040
4991	IGS2	2	2	0	YPS128 YPS606
5064	IGS2	5	2	3	UWOPS05-227-2 UWOPS03-461-4 DBVPG6040 UWOPS05-217-3 UWOPS83-787-3
5069	IGS2	33	27	6	L_1374 DBVPG1106 DBVPG1788 YJM975 YJM978 YPS128 YJM981 S288c NCYC110 YPS606 BC187 DBVPG6765 DBVPG6044 SK1 DBVPG1373 UWOPS87-2421 322134S Y55 273614N Y9 Y12 UWOPS05-227-2 378604X K11 UWOPS03-461-4 DBVPG6040 UWOPS05-217-3 YS9 YIIc17_E5 UWOPS83-787-3 NCYC361 YS4 DBVPG1853
5131	IGS2	6	2	4	UWOPS05-227-2 UWOPS03-461-4 DBVPG6040 UWOPS05-217-3 YIIc17_E5 UWOPS83-787-3
5142	IGS2	3	1	2	UWOPS87-2421 UWOPS83-787-3 NCYC361
5143	IGS2	4	0	4	322134S 378604X YS9 YS4
5195	IGS2	1	1	0	K11
5218	IGS2	5	3	2	UWOPS05-227-2 UWOPS03-461-4 DBVPG6040 UWOPS05-217-3 UWOPS83-787-3
5221	IGS2	1	0	1	NCYC361
5238	IGS2	31	22	9	L_1374 DBVPG1106 DBVPG1788 YJM975 YJM978 YPS128 YJM981 S288c NCYC110 YPS606 BC187 DBVPG6765 DBVPG6044 SK1 DBVPG1373 UWOPS87-2421 Y55 273614N Y9 Y12 UWOPS05-227-2 378604X K11 UWOPS03-461-4 DBVPG6040 UWOPS05-217-3 YS9 YIIc17_E5 UWOPS83-787-3 NCYC361 YS4
5248	IGS2	33	27	6	L_1374 DBVPG1106 DBVPG1788 YJM975 YJM978 YPS128 YJM981 S288c NCYC110 YPS606 BC187 DBVPG6765 DBVPG6044 SK1 DBVPG1373 UWOPS87-2421 322134S Y55 273614N Y9 Y12 UWOPS05-227-2 378604X K11 UWOPS03-461-4 DBVPG6040 UWOPS05-217-3 YS9 YIIc17_E5 UWOPS83-787-3 NCYC361 YS4 DBVPG1853
5260	IGS2	7	2	5	273614N Y9 Y12 K11 YS9 YIIc17_E5 YS4
5277	IGS2	4	4	0	NCYC110 DBVPG6044 SK1 Y55
5279	IGS2	2	0	2	YS9 YS4
5301	IGS2	5	3	2	UWOPS05-227-2 UWOPS03-461-4 DBVPG6040 UWOPS05-217-3 UWOPS83-787-3
5329	IGS2	33	27	6	L_1374 DBVPG1106 DBVPG1788 YJM975 YJM978 YPS128 YJM981 S288c NCYC110 YPS606 BC187 DBVPG6765 DBVPG6044 SK1 DBVPG1373 UWOPS87-2421 322134S Y55 273614N Y9 Y12 UWOPS05-227-2 378604X K11 UWOPS03-461-4 DBVPG6040 UWOPS05-217-3 YS9 YIIc17_E5 UWOPS83-787-3 NCYC361 YS4 DBVPG1853
5396	IGS2	5	3	2	UWOPS05-227-2 UWOPS03-461-4 DBVPG6040 UWOPS05-217-3 UWOPS83-787-3
5443	IGS2	1	0	1	DBVPG1853
5455	IGS2	2	1	1	322134S NCYC361
5457	IGS2	4	3	1	YPS128 YPS606 BC187 K11
5473	IGS2	1	0	1	YPS606
5499	IGS2	1	1	0	DBVPG1853
5524	IGS2	4	4	0	NCYC110 DBVPG6044 SK1 Y55
5526	IGS2	3	3	0	UWOPS05-227-2 UWOPS03-461-4 UWOPS05-217-3
5527	IGS2	3	3	0	UWOPS05-227-2 UWOPS03-461-4 UWOPS05-217-3
5554	IGS2	3	0	3	YJM975 YJM978 YJM981
5579	IGS2	1	0	1	S288c
5601	IGS2	3	0	3	W303 S288c YIIc17_E5
5602	IGS2	2	0	2	DBVPG6040 NCYC361
5659	IGS2	6	2	4	273614N DBVPG6040 YS9 NCYC361 YS4 378604X
5662	IGS2	3	1	2	Y9 Y12 K11
5667	IGS2	1	0	1	W303
5714	IGS2	2	1	1	BC187 DBVPG1853
5756	IGS2	4	1	3	273614N YS9 YS4 DBVPG1853

5792	IGS2	1	1	0	DBVPG6765																			
5818	IGS2	4	3	1	UWOPS05-227-2	UWOPS03-461-4	UWOPS05-217-3	UWOPS83-787-3																
5844	IGS2	1	1	0	DBVPG6765																			
5846	IGS2	1	0	1	DBVPG1853																			
5850	IGS2	1	0	1	YIIc17_E5																			
5853	IGS2	7	6	1	DBVPG6765	UWOPS87-2421	UWOPS05-227-2	UWOPS03-461-4	UWOPS05-217-3	UWOPS83-787-3	NCYC361													
5912	ETS1	1	1	0	DBVPG6765																			
5940	ETS1	1	0	1	DBVPG1373																			
5998	ETS1	1	0	1	378604X																			
6004	ETS1	2	0	2	322134S	NCYC361																		
6089	ETS1	20	15	5	YPS128	S288c	NCYC110	YPS606	DBVPG6765	DBVPG6044	SK1	UWOPS87-2421	Y55	273614N	Y9	Y12	UWOPS05-227-2	K11	UWOPS03-461-4	DBVPG6040	UWOPS05-217-3	YIIc17_E5	UWOPS83-787-3	NCYC361
6295	ETS1	3	1	2	Y9	Y12	K11																	
6479	ETS1	1	0	1	DBVPG1373																			
6514	ETS1	3	0	3	378604X	YS9	YS4																	
6521	ETS1	1	0	1	DBVPG1853																			
6528	ETS1	2	2	0	DBVPG1106	UWOPS87-2421																		
6540	ETS1	1	0	1	DBVPG1853																			
6548	ETS1	2	2	0	DBVPG1373	UWOPS05-227-2																		
6549	ETS1	3	2	1	DBVPG1373	UWOPS05-227-2	DBVPG1853																	
6568	ETS1	1	1	0	K11																			
6595	18S	1	0	1	YJM975																			
7322	18S	5	0	5	273614N	378604X	YIIc17_E5	YS4	DBVPG1853															
7881	18S	1	0	1	BC187																			
8295	18S	2	0	2	Y9	Y12																		
8412	ITS1	4	4	0	NCYC110	DBVPG6044	SK1	Y55																
8462	ITS1	1	0	1	DBVPG1373																			
8505	ITS1	3	2	1	DBVPG1788	DBVPG6765	YIIc17_E5																	
8546	ITS1	1	0	1	DBVPG1853																			
8568	ITS1	4	0	4	NCYC110	DBVPG6044	SK1	Y55																
8652	ITS1	1	0	1	DBVPG1853																			
8686	ITS1	18	13	5	YPS128	NCYC110	YPS606	DBVPG6044	SK1	UWOPS87-2421	Y55	Y9	Y12	UWOPS05-227-2	K11	UWOPS03-461-4	DBVPG6040	UWOPS05-217-3	YIIc17_E5	UWOPS83-787-3	NCYC361	YS4		
8692	ITS1	1	0	1	DBVPG6040																			
8702	ITS1	2	0	2	YS9	YS4																		
8738	ITS1	2	0	2	DBVPG6040	YIIc17_E5																		
8990	ITS2	1	0	1	K11																			
9026	ITS2	1	0	1	YJM978																			
9027	ITS2	1	0	1	YJM978																			
		**650**	**339**	**311**																				

Supplementary Table S2: pSNP frequencies in *Saccharomyces cerevisiae* strains

Position	Region	pSNPs	Strains												Reference	Variants	Frequencies
524	26S	2	DBVPG6040	NCYC361											81	10	0.110
															91	2	0.022
679	26S	1	DBVPG6044												132	6	0.043
1112	26S	1	UWOPS87-2421												35	2	0.054
1426	26S	1	DBVPG1373												45	21	0.318
1813	26S	1	YS9					Supplementary Table S2: pSNP frequencies in Saccharomyces paradoxus strains							40	2	0.048
1852	26S	1	DBVPG6765												182	10	0.052
1887	26S	1	DBVPG1853												89	24	0.212
2017	26S	1	YJM981												200	5	0.024
2420	26S	1	DBVPG1373												75	3	0.038
2996	26S	1	YPS606												86	3	0.034
3012	26S	1	DBVPG6765												177	19	0.097
3096	26S	1	273614N												68	2	0.029
3154	26S	1	322134S												100	2	0.020
3177	26S	3	NCYC110	SK1	Y55										119	9	0.070
															236	9	0.037
															237	8	0.033
3266	26S	1	DBVPG1853												4	94	0.959
3430	ETS2	1	YS4												38	4	0.095
3494	ETS2	2	YS9	YS4											6	31	0.838
															6	46	0.885
3517	ETS2	3	UWOPS87-2421	UWOPS05-217-3	UWOPS83-787-3										52	2	0.037
															2	121	0.984
															55	7	0.113
3538	ETS2	2	Y9	Y12											11	45	0.804
															23	35	0.603
3555	ETS2	2	Y9	Y12											11	38	0.776
															23	33	0.589
3590	ETS2	1	NCYC361												82	6	0.068
3599	ETS2	1	NCYC361												81	6	0.069
3609	IGS1	1	Y55												185	3	0.016
3610	IGS1	1	Y55												185	3	0.016
3611	IGS1	1	Y55												185	3	0.016
3612	IGS1	2	Y55	YIIc17_E5											185	3	0.016
															44	2	0.043
3624	IGS1	1	YIIc17_E5												17	12	0.414
3659	IGS1	12	DBVPG1106	YJM975	YJM978	YJM981	BC187	DBVPG1373	273614N	378604X	DBVPG6040	YIIc17_E5	NCYC361	YS4	16	17	0.515
															16	35	0.686
															17	18	0.514
															59	84	0.587
															12	14	0.538
															29	43	0.597
															7	29	0.806
															62	5	0.075
															79	5	0.060
															10	41	0.804
															8	57	0.877
															5	36	0.878
3678	IGS1	2	YS9	YS4											3	29	0.906
															8	45	0.849
3687	IGS1	1	BC187												29	8	0.216
3697	IGS1	5	S288c	Y9	Y12	DBVPG6040	YIIc17_E5								108	17	0.136
															41	6	0.128

												42	11	0.208
												90	7	0.072
												52	2	0.037
3713	IGS1	2	UWOPS87-2421	UWOPS83-787-3								47	2	0.041
												43	7	0.140
3769	IGS1	1	YS9									6	42	0.875
3813	IGS1	5	273614N	378604X	YIIc17_E5	NCYC361	DBVPG1853					6	32	0.842
												11	40	0.784
												27	12	0.308
												13	47	0.783
												83	26	0.239
3840	IGS1	1	S288c									101	8	0.073
3871	IGS1	1	BC187									23	2	0.080
3902	IGS1	5	S288c	378604X	DBVPG6040	YS9	NCYC361					102	10	0.089
												2	37	0.949
												57	2	0.034
												3	26	0.897
												5	53	0.914
3903	IGS1	2	YS9	YS4								18	11	0.379
												18	27	0.600
3967	IGS1	1	Y55									252	2	0.008
3974	IGS1	6	UWOPS87-2421	322134S	Y9	Y12	UWOPS83-787-3	NCYC361				2	23	0.920
												6	63	0.913
												5	28	0.848
												12	23	0.657
												5	24	0.828
												8	50	0.862
3989	IGS1	4	322134S	DBVPG6040	YS9	YS4						76	2	0.026
												79	2	0.025
												29	27	0.482
												32	43	0.573
4056	IGS1	1	YIIc17_E5									14	20	0.588
4058	IGS1	1	YIIc17_E5									14	20	0.588
4070	IGS1	1	YS4									35	8	0.186
4129	IGS1	1	YS4									28	2	0.067
4156	IGS1	6	322134S	273614N	378604X	YS9	NCYC361	YS4				4	69	0.945
												20	31	0.608
												14	46	0.767
												11	28	0.718
												8	51	0.864
												4	60	0.938
4166	IGS1	9	S288c	322134S	273614N	378604X	DBVPG6040	YS9	YIIc17_E5	NCYC361	YS4	94	9	0.087
												3	66	0.957
												20	28	0.583
												13	38	0.745
												47	20	0.299
												10	27	0.730
												15	18	0.545
												4	43	0.915
												3	54	0.947
4183	IGS1	2	Y9	Y12								3	24	0.889
												10	16	0.615
4203	IGS1	3	322134S	YS9	NCYC361							5	84	0.944
												10	38	0.792
												5	58	0.921
4215	IGS1	7	S288c	322134S	273614N	378604X	DBVPG6040	YIIc17_E5	NCYC361			66	10	0.132

													66	3	0.043
													18	25	0.581
													12	46	0.793
													48	19	0.284
													11	21	0.656
													40	4	0.091
4224	IGS1	2	YS9	YS4									21	10	0.323
													20	30	0.600
4241	IGS1	1	YS4										46	11	0.193
4248	IGS1	5	322134S	273614N	378604X	YS9	NCYC361						4	67	0.944
													36	2	0.053
													11	42	0.792
													8	34	0.810
													5	45	0.900
4270	IGS1	1	UWOPS83-787-3										29	8	0.216
4284	IGS1	1	UWOPS83-787-3										18	6	0.250
4305	IGS1	1	DBVPG6040										45	12	0.211
4307	IGS1	1	S288c										90	4	0.043
4365	IGS1	1	UWOPS83-787-3										30	9	0.231
4386	IGS1	1	UWOPS83-787-3										30	9	0.231
4401	IGS1	1	DBVPG1853										51	17	0.250
4418	IGS1	1	Y12										39	4	0.093
4431	IGS1	1	W303										189	8	0.041
4484	IGS1	10	L_1374	YJM975	YJM981	BC187	SK1	Y12	K11	YS9	NCYC361	YS4	30	4	0.118
													33	2	0.057
													177	4	0.022
													26	2	0.071
													161	6	0.036
													22	4	0.154
													16	2	0.111
													26	3	0.103
													51	3	0.056
													42	4	0.087
4495	IGS1	1	DBVPG1853										38	19	0.333
4496	IGS1	4	UWOPS05-217-3	YS9	YS4	DBVPG1853							3	71	0.959
													22	7	0.241
													31	16	0.340
													21	40	0.656
4498	IGS1	3	273614N	YS9	YS4								22	18	0.450
													22	7	0.241
													31	16	0.340
4499	IGS1	1	DBVPG1853										41	20	0.328
4508	IGS1	1	DBVPG1373										24	16	0.400
4652	IGS2	1	DBVPG6765										133	58	0.304
4657	IGS2	1	L_1374										42	10	0.192
4664	IGS2	2	322134S	378604X									89	3	0.033
													19	42	0.689
4701	IGS2	3	UWOPS05-227-2	UWOPS05-217-3	UWOPS83-787-3								3	51	0.944
													2	92	0.979
													38	8	0.174
4728	IGS2	1	DBVPG1853										38	40	0.513
4754	IGS2	3	S288c	378604X	DBVPG6040								105	8	0.071
													58	15	0.205
													70	14	0.167
4763	IGS2	2	273614N	378604X									26	29	0.527
													65	2	0.030

4769	IGS2	5	322134S	UWOPS05-227-2	378604X	YS9	UWOPS83-787-3					81	3	0.036
												3	57	0.950
												18	52	0.743
												2	29	0.935
												42	9	0.176
4786	IGS2	1	BC187									27	2	0.069
4809	IGS2	1	DBVPG6040									83	3	0.035
4854	IGS2	4	UWOPS05-227-2	DBVPG6040	UWOPS83-787-3	NCYC361						3	54	0.947
												91	2	0.022
												3	37	0.925
												68	6	0.081
4925	IGS2	2	UWOPS05-227-2	UWOPS83-787-3								3	53	0.946
												46	8	0.148
4928	IGS2	2	UWOPS05-227-2	UWOPS83-787-3								3	56	0.949
												46	7	0.132
4963	IGS2	1	DBVPG6040									92	3	0.032
5064	IGS2	3	UWOPS05-227-2	DBVPG6040	UWOPS83-787-3							2	40	0.952
												90	2	0.022
												39	7	0.152
5069	IGS2	6	S288c	273614N	378604X	DBVPG6040	YS9	NCYC361				110	7	0.060
												32	26	0.448
												13	40	0.755
												72	17	0.191
												3	25	0.893
												6	54	0.900
5131	IGS2	4	UWOPS05-227-2	DBVPG6040	YIIc17_E5	UWOPS83-787-3						3	44	0.936
												89	2	0.022
												2	43	0.956
												35	4	0.103
5142	IGS2	2	UWOPS83-787-3	NCYC361								8	35	0.814
												69	7	0.092
5143	IGS2	4	322134S	378604X	YS9	YS4						78	2	0.025
												14	39	0.736
												18	14	0.438
												19	25	0.568
5218	IGS2	2	DBVPG6040	UWOPS83-787-3								82	2	0.024
												32	5	0.135
5221	IGS2	1	NCYC361									62	6	0.088
5238	IGS2	9	S288c	273614N	Y9	Y12	378604X	DBVPG6040	YS9	NCYC361	YS4	92	6	0.061
												34	30	0.469
												17	8	0.320
												18	7	0.280
												55	2	0.035
												68	15	0.181
												22	14	0.389
												58	10	0.147
												31	6	0.162
5248	IGS2	6	S288c	273614N	378604X	DBVPG6040	YS9	NCYC361				91	8	0.081
												34	29	0.460
												11	36	0.766
												70	15	0.176
												4	32	0.889
												3	69	0.958
5260	IGS2	5	273614N	Y9	YS9	YIIc17_E5	YS4					34	29	0.460
												3	17	0.850
												18	22	0.550

									2	42	0.955
									32	7	0.179
5279	IGS2	2	YS9	YS4					4	28	0.875
									10	28	0.737
5301	IGS2	2	DBVPG6040	UWOPS83-787-3					84	2	0.023
									36	3	0.077
5329	IGS2	6	S288c	273614N	378604X	DBVPG6040	YS9	NCYC361	84	8	0.087
									32	27	0.458
									12	32	0.727
									69	15	0.179
									2	26	0.929
									3	60	0.952
5396	IGS2	2	DBVPG6040	UWOPS83-787-3					87	2	0.022
									42	2	0.045
5443	IGS2	1	DBVPG1853						108	12	0.100
5455	IGS2	1	NCYC361						13	56	0.812
5457	IGS2	1	BC187						27	2	0.069
5473	IGS2	1	YPS606						65	4	0.058
5554	IGS2	3	YJM975	YJM978	YJM981				32	5	0.135
									23	13	0.361
									103	53	0.340
5579	IGS2	1	S288c						69	8	0.104
5601	IGS2	3	W303	S288c	YIIc17_E5				276	3	0.011
									77	8	0.094
									44	3	0.064
5602	IGS2	2	DBVPG6040	NCYC361					51	11	0.177
									8	44	0.846
5659	IGS2	4	DBVPG6040	YS9	NCYC361	378604X			53	11	0.172
									2	32	0.941
									50	2	0.038
									8	39	0.830
5662	IGS2	2	Y9	Y12					3	16	0.842
									13	18	0.581
5667	IGS2	1	W303						273	3	0.011
5714	IGS2	1	DBVPG1853						59	31	0.344
5756	IGS2	3	YS9	YS4	DBVPG1853				26	11	0.297
									35	28	0.444
									33	70	0.680
5818	IGS2	1	UWOPS83-787-3						24	4	0.143
5846	IGS2	1	DBVPG1853						107	3	0.027
5850	IGS2	1	YIIc17_E5						4	55	0.932
5853	IGS2	1	NCYC361						76	7	0.084
5940	ETS1	1	DBVPG1373						70	12	0.146
5998	ETS1	1	378604X						9	49	0.845
6004	ETS1	2	322134S	NCYC361					3	51	0.944
									17	59	0.776
6089	ETS1	5	S288c	DBVPG6040	YIIc17_E5	UWOPS83-787	NCYC361		99	6	0.057
									55	12	0.179
									4	57	0.934
									2	23	0.920
									72	13	0.153
6295	ETS1	2	Y9	Y12					7	30	0.811
									12	29	0.707
6479	ETS1	1	DBVPG1373						85	2	0.023
6514	ETS1	3	378604X	YS9	YS4				8	25	0.758
									17	4	0.190

								23	34	0.596
6521	ETS1	1	DBVPG1853					36	48	0.571
6540	ETS1	1	DBVPG1853					77	4	0.049
6549	ETS1	1	DBVPG1853					36	46	0.561
6595	18S	1	YJM975					29	2	0.065
7322	18S	5	273614N	378604X	YIIc17_E5	YS4	DBVPG1853	65	2	0.030
								16	37	0.698
								2	37	0.949
								54	9	0.143
								72	16	0.182
7881	18S	1	BC187					30	2	0.063
8295	18S	2	Y9	Y12				5	28	0.848
								21	25	0.543
8462	ITS1	1	DBVPG1373					77	2	0.025
8505	ITS1	1	YIIc17_E5					49	12	0.197
8546	ITS1	1	DBVPG1853					87	21	0.194
8568	ITS1	4	NCYC110	DBVPG6044	SK1	Y55		42	37	0.468
								93	16	0.147
								28	147	0.840
								74	122	0.622
8652	ITS1	1	DBVPG1853					56	17	0.233
8686	ITS1	5	DBVPG6040	YIIc17_E5	UWOPS83-787-3	NCYC361	YS4	63	28	0.308
								15	37	0.712
								2	36	0.947
								80	9	0.101
								38	21	0.356
8692	ITS1	1	DBVPG6040					58	26	0.310
8702	ITS1	2	YS9	YS4				28	24	0.462
								50	17	0.254
8738	ITS1	2	DBVPG6040	YIIc17_E5				55	26	0.321
								15	40	0.727
8990	ITS2	1	K11					2	17	0.895
9026	ITS2	1	YJM978					31	11	0.262
9027	ITS2	1	YJM978					32	11	0.256
		**311**								

Supplementary Table S2: INSs and pINSs in *Saccharomyces cerevisiae* strains

Position	Region	No. of strains	INSs	pINSs	Type 1 pINSs	Type 2 pINSs	Strains						
547	26S	2	0	2	2	0	UWOPS83-787-3	NCYC361					
3625	IGS1	1	0	1	0	1	YS9						
3626	IGS1	5	0	5	2	3	273614N	378604X	DBVPG6040	Y9	YIIc17_E5		
3629	IGS1	1	0	1	0	1	DBVPG1853						
3630	IGS1	3	1	2	1	1	NCYC110	BC187	YS4				
3631	IGS1	2	1	1	0	1	Y55	Y12					
3632	IGS1	1	1	0	0	0	DBVPG6044						
3702	IGS1	1	0	1	1	0	YIIc17_E5						
3901	IGS1	1	0	1	0	1	DBVPG1853						
3902	IGS1	1	0	1	0	1	Y12						
3903	IGS1	1	1	0	0	0	K11						
4049	IGS1	5	1	4	4	0	S288c	322134S	378604X	YS9	YS4		
4051	IGS1	1	1	0	0	0	K11						
4052	IGS1	1	1	0	0	0	Y12						
4054	IGS1	1	0	1	1	0	NCYC361						
4056	IGS1	2	1	1	1	0	Y9	DBVPG6040					
4059	IGS1	1	0	1	1	0	YIIc17_E5						
4312	IGS1	1	1	0	0	0	K11						
4315	IGS1	1	1	0	0	0	K11						
4319	IGS1	1	1	0	0	0	K11						
5622	IGS2	2	1	1	1	0	273614N	378604X					
5632	IGS2	7	2	5	3	2	NCYC110	DBVPG1373	Y9	YIIc17_E5	DBVPG1853	NCYC361	Y12
5643	IGS2	1	0	1	1	0	YS4						
5644	IGS2	1	0	1	1	0	DBVPG6044						
5653	IGS2	1	0	1	1	0	DBVPG1853						
5707	IGS2	1	1	0	0	0	273614N						
8413	ITS1	2	0	2	1	1	DBVPG1853	NCYC361					
8414	ITS1	1	0	1	0	1	YS9						
8652	ITS1	1	0	1	1	0	378604X						
8665	ITS1	1	0	1	1	0	YS4						
8982	ITS2	4	0	4	3	1	322134S	YS9	DBVPG1853	YS4			
9031	ITS2	2	0	2	2	0	Y9	Y12					
		**57**	**15**	**42**	**28**	**14**							

Supplementary Table S2: pINS frequencies in *Saccharomyces cerevisiae* strains

Position	Region	Type 1 pINSs	Strains				Reference	Variants	Frequencies
547	26S	2	UWOPS83-787-3	NCYC361			26	9	0.257
							91	2	0.022
3626	IGS1	2	273614N	DBVPG6040			29	2	0.065
							75	2	0.026
3630	IGS1	1	BC187				19	8	0.296
3702	IGS1	1	YIIc17_E5				24	28	0.538
4049	IGS1	4	S288c	322134S	378604X	YS9	106	10	0.086
							6	39	0.867
							14	28	0.667
							9	27	0.750
4054	IGS1	1	NCYC361				2	41	0.953
4056	IGS1	1	DBVPG6040				5	36	0.878
4059	IGS1	1	YIIc17_E5				14	20	0.588
5622	IGS2	1	378604X				8	25	0.758
5632	IGS2	3	DBVPG1373	Y9	YIIc17_E5		37	8	0.178
							5	12	0.706
							6	34	0.850
5643	IGS2	1	YS4				43	4	0.085
5644	IGS2	1	DBVPG6044				3	70	0.959
5653	IGS2	1	DBVPG1853				85	28	0.248
8413	ITS1	1	DBVPG1853				18	57	0.760
8652	ITS1	1	378604X				16	33	0.673
8665	ITS1	1	YS4				20	35	0.636
8982	ITS2	3	322134S	YS9	DBVPG1853		77	3	0.038
							7	34	0.829
							30	71	0.703
9031	ITS2	2	Y9	Y12			10	26	0.722
							8	41	0.837
		**28**							

Supplementary Table S2: DELs and pDELs in *Saccharomyces cerevisiae* strains

Start position	End position	Region	No. of strains	DELs	pDELs	Type 1 pDELs	Type 2 pDELs	Strains
309	309	26S	1	0	1	1	0	DBVPG6765
3543	3543	ETS2	1	0	1	1	0	SK1
3578	3601	ETS2	3	2	1	1	0	YS9 YS4 DBVPG1853
3580	3603	ETS2	1	0	1	1	0	378604X
3592	3597	ETS2	1	0	1	1	0	NCYC361
3593	3599	ETS2	5	3	2	2	0	UWOPS87-2421 273614N UWOPS05-227-2 UWOPS05-217-3 UWOPS83-787-3
3595	3600	ETS2	1	0	1	1	0	Y9
3595	3608	ETS2	1	0	1	1	0	YIIc17_E5
3597	3605	ETS2	1	0	1	1	0	DBVPG6040
3599	3599	ETS2	1	1	0	0	0	UWOPS03-461-4
3600	3602	ETS2	1	0	1	0	1	Y55
3600	3605	ETS2	1	0	1	1	0	NCYC361
3601	3607	ETS2	2	1	1	1	0	NCYC110 273614N
3602	3608	ETS2	2	2	0	0	0	Y12 K11
3619	3629	IGS1	1	0	1	0	1	K11
3626	3627	IGS1	1	1	0	0	0	DBVPG1106
3626	3628	IGS1	6	5	1	1	0	DBVPG1788 DBVPG6765 DBVPG1373 UWOPS05-227-2 UWOPS03-461-4 UWOPS05-217-3
3626	3629	IGS1	8	2	6	4	2	YPS128 YPS606 YJM975 YJM978 YJM981 UWOPS87-2421 UWOPS83-787-3 NCYC361
3627	3627	IGS1	1	0	1	1	0	273614N
3627	3628	IGS1	1	0	1	1	0	YIIc17_E5
3627	3629	IGS1	1	0	1	1	0	BC187
3640	3640	IGS1	1	1	0	0	0	YPS606
3833	3833	IGS1	27	23	4	4	0	L_1374 DBVPG1106 DBVPG1788 YJM975 YJM978 YJM981 NCYC110 YPS606 BC187 DBVPG6765 DBVPG6044 SK1 DBVPG1373 UWOPS87-2421 322134S Y55 273614N Y9 Y12 UWOPS05-227-2 378604X K11 DBVPG6040 UWOPS05-217-3 UWOPS83-787-3 NCYC361 DBVPG1853
3834	3834	IGS1	1	1	0	0	0	UWOPS03-461-4
3835	3835	IGS1	1	1	0	0	0	YPS128
3837	3837	IGS1	1	0	1	1	0	S288c
3840	3840	IGS1	1	0	1	1	0	DBVPG6765
3841	3841	IGS1	1	0	1	1	0	S288c
3842	3842	IGS1	1	1	0	0	0	YIIc17_E5
3881	3881	IGS1	1	1	0	0	0	UWOPS03-461-4
3882	3888	IGS1	1	0	1	1	0	Y12
3883	3888	IGS1	1	1	0	0	0	UWOPS03-461-4
3893	3900	IGS1	4	2	2	1	1	UWOPS05-227-2 UWOPS05-217-3 YS9 YS4
3894	3901	IGS1	1	0	1	0	1	UWOPS83-787-3
3900	3900	IGS1	24	20	4	4	0	L_1374 DBVPG1106 DBVPG1788 YJM975 YJM978 YPS128 YJM981 S288c NCYC110 YPS606 BC187 DBVPG6765 DBVPG6044 SK1 DBVPG1373 UWOPS87-2421 322134S Y55 273614N 378604X UWOPS03-461-4 YIIc17_E5 NCYC361 DBVPG1853
3901	3901	IGS1	2	0	2	2	0	Y12 DBVPG6040
3913	3919	IGS1	2	2	0	0	0	K11 UWOPS03-461-4
3913	3920	IGS1	1	0	1	0	1	YS4
3913	3921	IGS1	1	0	1	0	1	DBVPG1853
3913	3922	IGS1	9	4	5	0	5	L_1374 DBVPG1106 YJM975 YJM978 YJM981 DBVPG1373 273614N 378604X DBVPG6040
3913	3923	IGS1	6	4	2	0	2	DBVPG1788 YPS128 YPS606 DBVPG6765 BC187 YIIc17_E5
3913	3924	IGS1	5	2	3	0	3	DBVPG6044 UWOPS87-2421 Y9 Y12 NCYC361
3913	3925	IGS1	1	1	0	0	0	NCYC110
3913	3926	IGS1	3	2	1	0	1	UWOPS05-227-2 UWOPS05-217-3 UWOPS83-787-3
3920	3921	IGS1	1	0	1	1	0	YS9
3920	3922	IGS1	1	1	0	0	0	322134S
3920	3924	IGS1	3	2	1	1	0	S288c SK1 Y55
3921	3934	IGS1	1	1	0	0	0	UWOPS03-461-4
3934	3934	IGS1	1	1	0	0	0	YS4
4101	4115	IGS1	2	2	0	0	0	UWOPS87-2421 DBVPG1853
4101	4116	IGS1	26	21	5	5	0	L_1374 DBVPG1106 DBVPG1788 YJM975 YJM978 YPS128 YJM981 NCYC110 BC187 DBVPG6765 DBVPG6044 322134S Y55 Y9 Y12 UWOPS05-227-2 378604X K11 UWOPS03-461-4 DBVPG6040 UWOPS05-217-3 YS9 YIIc17_E5 UWOPS83-787-3 NCYC361 YS4
4104	4119	IGS1	1	1	0	0	0	DBVPG1373
4106	4121	IGS1	1	0	1	0	1	YPS606
4108	4119	IGS1	1	1	0	0	0	SK1
4109	4119	IGS1	1	0	1	1	0	S288c
4240	4259	IGS1	2	0	2	2	0	YJM975 YJM978
4244	4259	IGS1	1	0	1	1	0	YJM981
4298	4307	IGS1	1	1	0	0	0	YJM975
4299	4299	IGS1	2	2	0	0	0	378604X K11
4299	4301	IGS1	1	0	1	0	1	YS9
4299	4303	IGS1	4	1	3	0	3	322134S Y9 YS4 DBVPG1853
4299	4305	IGS1	1	0	1	1	0	NCYC361
4299	4306	IGS1	4	2	2	0	2	DBVPG1788 YPS606 DBVPG6765 YIIc17_E5
4299	4307	IGS1	11	5	6	0	6	L_1374 DBVPG1106 YJM978 YJM981 BC187 SK1 DBVPG1373 UWOPS87-2421 Y55 273614N Y12
4299	4308	IGS1	2	1	1	0	1	UWOPS05-227-2 UWOPS05-217-3
4300	4303	IGS1	1	0	1	1	0	DBVPG6040
4300	4306	IGS1	2	1	1	1	0	YPS128 S288c
4300	4307	IGS1	2	2	0	0	0	NCYC110 DBVPG6044
4300	4308	IGS1	2	1	1	0	1	UWOPS03-461-4 UWOPS83-787-3
4309	4312	IGS1	1	0	1	0	1	378604X
4484	4494	IGS1	1	0	1	0	1	UWOPS05-217-3
4484	4496	IGS1	1	0	1	0	1	273614N
4486	4492	IGS1	1	0	1	1	0	378604X
4486	4494	IGS1	2	0	2	0	2	YS9 YS4
4486	4495	IGS1	7	0	7	2	5	L_1374 YJM975 YJM978 YJM981 DBVPG1373 K11 DBVPG6040
4486	4496	IGS1	4	1	3	0	3	DBVPG1106 322134S Y9 UWOPS03-461-4
4486	4497	IGS1	1	0	1	1	0	SK1
4486	4498	IGS1	8	2	6	1	5	DBVPG1788 YPS128 DBVPG6765 BC187 YPS606 DBVPG6044 YIIc17_E5 NCYC361
4486	4499	IGS1	2	1	1	0	1	UWOPS87-2421 UWOPS83-787-3
4498	4510	IGS1	1	0	1	1	0	S288c
4500	4505	IGS1	1	0	1	0	1	DBVPG1853
4500	4508	IGS1	1	1	0	0	0	DBVPG1106
4511	4512	IGS1	1	0	1	1	0	273614N
5221	5221	IGS2	2	1	1	1	0	UWOPS87-2421 UWOPS83-787-3
5222	5222	IGS2	1	0	1	1	0	NCYC361
5585	5585	IGS2	3	2	1	1	0	UWOPS05-227-2 UWOPS03-461-4 UWOPS05-217-3
5594	5599	IGS2	2	0	2	2	0	378604X DBVPG6040
5594	5602	IGS2	3	2	1	1	0	322134S 273614N YS9
5600	5606	IGS2	1	1	0	0	0	DBVPG1853
5602	5610	IGS2	1	1	0	0	0	YS4
5605	5613	IGS2	1	0	1	1	0	378604X
5665	5665	IGS2	1	0	1	1	0	W303
5667	5667	IGS2	5	3	2	2	0	S288c Y9 Y12 K11 YIIc17_E5
5699	5699	IGS2	1	1	0	0	0	YS4
6520	6520	ETS1	1	0	1	1	0	DBVPG1853
6521	6521	ETS1	2	0	2	2	0	YS9 YS4
6521	6523	ETS1	5	4	1	0	1	322134S Y55 273614N K11 YIIc17_E5
6521	6524	ETS1	22	16	6	2	4	L_1374 DBVPG1788 YJM975 YJM978 YPS128 YJM981 S288c NCYC110 YPS606 BC187 DBVPG6765 DBVPG6044 SK1 DBVPG1373 Y9 Y12 UWOPS05-227-2 378604X UWOPS03-461-4 UWOPS05-217-3 UWOPS83-787-3 NCYC361
6528	6531	ETS1	1	0	1	1	0	DBVPG6040
6530	6530	ETS1	1	1	0	0	0	Y55
6530	6533	ETS1	2	2	0	0	0	DBVPG1106 UWOPS87-2421
6534	6534	ETS1	1	0	1	1	0	DBVPG1853
6543	6545	ETS1	2	2	0	0	0	DBVPG1373 UWOPS05-227-2
6546	6546	ETS1	5	5	0	0	0	DBVPG1788 YJM981 DBVPG6044 Y55 UWOPS05-217-3
6547	6549	ETS1	20	18	2	2	0	L_1374 DBVPG1106 YJM975 YJM978 YPS128 S288c NCYC110 YPS606 BC187 DBVPG6765 SK1 UWOPS87-2421 322134S 273614N Y9 Y12 K11 UWOPS03-461-4 UWOPS83-787-3 NCYC361
6548	6549	ETS1	6	6	0	0	0	DBVPG1788 YJM981 DBVPG6044 Y55 UWOPS05-217-3 YIIc17_E5
6548	6550	ETS1	2	0	2	2	0	378604X DBVPG6040
8013	8013	18S	1	0	1	1	0	UWOPS83-787-3
9067	9067	ITS2	23	15	8	8	0	DBVPG1788 YPS128 NCYC110 YPS606 BC187 DBVPG6765 DBVPG6044 SK1 UWOPS87-2421 322134S Y55 Y9 Y12 UWOPS05-227-2 UWOPS03-461-4 DBVPG6040 UWOPS05-217-3 YS9 YIIc17_E5 UWOPS83-787-3 NCYC361 YS4 DBVPG1853
9068	9068	ITS2	1	1	0	0	0	K11
			**360**	**215**	**145**	**86**	**59**	

Supplementary Table S2: pDEL frequencies in *Saccharomyces cerevisiae* strains

Start position	End position	Region	Type 1 pDELs	Strains					Reference	Variants	Frequencies
309	309	26S	1	DBVPG6765					242	2	0.008
3543	3543	ETS2	1	SK1					252	2	0.008
3578	3601	ETS2	1	DBVPG1853					45	75	0.625
3580	3603	ETS2	1	378604X					60	8	0.118
3592	3597	ETS2	1	NCYC361					79	6	0.071
3593	3599	ETS2	2	273614N	UWOPS05-217-3				59	3	0.048
									3	84	0.966
3595	3600	ETS2	1	Y9					5	39	0.886
3595	3608	ETS2	1	**YIIc17_E5**					28	22	0.440
3597	3605	ETS2	1	DBVPG6040					82	4	0.047
3600	3605	ETS2	1	NCYC361					79	8	0.092
3601	3607	ETS2	1	273614N					28	6	0.176
3626	3628	IGS1	1	**DBVPG1373**					40	40	0.500
3626	3629	IGS1	4	**YJM975**	**YJM978**	**YJM981**	NCYC361		30	25	0.455
									23	21	0.477
									105	79	0.429
									68	6	0.081
3627	3627	IGS1	1	273614N					3	28	0.903
3627	3628	IGS1	1	YIIc17_E5					17	13	0.433
3627	3629	IGS1	1	BC187					9	18	0.667
3833	3833	IGS1	4	378604X	DBVPG6040	NCYC361	DBVPG1853		9	43	0.827
									78	5	0.060
									7	46	0.868
									80	20	0.200
3837	3837	IGS1	1	S288c					113	9	0.074
3840	3840	IGS1	1	DBVPG6765					2	134	0.985
3841	3841	IGS1	1	S288c					99	8	0.075
3882	3888	IGS1	1	Y12					16	13	0.448
3893	3900	IGS1	1	**YS9**					17	17	0.500
3900	3900	IGS1	4	S288c	378604X	NCYC361	DBVPG1853		104	10	0.088
									2	37	0.949
									5	53	0.914
									15	64	0.810
3901	3901	IGS1	2	Y12	DBVPG6040				20	12	0.375
									57	2	0.034
3920	3921	IGS1	1	YS9					4	13	0.765
3920	3924	IGS1	1	S288c					117	10	0.079
4101	4116	IGS1	5	322134S	378604X	DBVPG6040	YS9	NCYC361	2	60	0.968
									14	35	0.714
									69	2	0.028
									10	31	0.756
									8	52	0.867
4109	4119	IGS1	1	S288c					98	8	0.075
4240	4259	IGS1	2	YJM975	YJM978				34	5	0.128
									17	17	0.500
4244	4259	IGS1	1	YJM981					112	45	0.287
4299	4305	IGS1	1	**NCYC361**					11	23	0.676
4300	4303	IGS1	1	DBVPG6040					43	13	0.232

4300	4306	IGS1	1	**S288c**								86	4	0.044
4486	4492	IGS1	1	378604X								2	13	0.867
4486	4495	IGS1	2	YJM981	K11							5	192	0.975
												5	14	0.737
4486	4497	IGS1	1	SK1								4	167	0.977
4486	4498	IGS1	1	**YIIc17_E5**								8	28	0.778
4498	4510	IGS1	1	S288c								79	4	0.048
4511	4512	IGS1	1	273614N								25	12	0.324
5221	5221	IGS2	1	UWOPS83-787-3								4	36	0.900
5222	5222	IGS2	1	NCYC361								61	6	0.090
5585	5585	IGS2	1	UWOPS05-217-3								2	51	0.962
5594	5599	IGS2	2	378604X	DBVPG6040							9	21	0.700
												53	10	0.159
5594	5602	IGS2	1	YS9								2	26	0.929
5605	5613	IGS2	1	378604X								10	31	0.756
5665	5665	IGS2	1	W303								277	3	0.011
5667	5667	IGS2	2	S288c	YIIc17_E5							73	6	0.076
												4	40	0.909
6520	6520	ETS1	1	DBVPG1853								36	48	0.571
6521	6521	ETS1	2	YS9	YS4							15	7	0.318
												24	32	0.571
6521	6524	ETS1	2	S288c	**NCYC361**							107	3	0.027
												31	29	0.483
6528	6531	ETS1	1	DBVPG6040								65	7	0.097
6534	6534	ETS1	1	DBVPG1853								27	58	0.682
6547	6549	ETS1	2	S288c	NCYC361							110	3	0.027
												8	49	0.860
6548	6550	ETS1	2	378604X	DBVPG6040							28	6	0.176
												65	7	0.097
8013	8013	18S	1	UWOPS83-787-3								55	2	0.035
9067	9067	ITS2	8	NCYC110	BC187	322134S	DBVPG6040	UWOPS05-217-3	YS9	NCYC361	DBVPG1853	2	86	0.977
												12	21	0.636
												87	3	0.033
												47	24	0.338
												2	67	0.971
												6	34	0.850
												2	67	0.971
												38	71	0.651
			**86**											

Supplementary Table S2: Complex variants in *Saccharomyces cerevisiae* strains

Strain	Start position	End position	Region	Details
Y9	3901	3904	IGS1	Possibly a pDEL + pINS, with sequence aca replaced by ta in 5 out of 27 reads

Supplementary Table S2: All variants in *Saccharomyces cerevisiae* strains

Strain	SNPs	pSNPs	INSs	pINSs	DELs	pDELs	CX	Total
W303	0	3	0	0	0	1	0	**4**
L_1374	6	2	0	0	7	1	0	**16**
DBVPG1106	7	1	0	0	9	1	0	**18**
DBVPG1788	8	0	0	0	11	0	0	**19**
YJM975	6	4	0	0	7	3	0	**20**
YJM978	6	4	0	0	7	3	0	**20**
YJM981	6	4	0	0	6	5	0	**21**
YPS128	14	0	0	0	10	0	0	**24**
S288c	0	14	0	1	0	10	0	**25**
BC187	7	7	0	1	5	5	0	**25**
DBVPG1373	8	7	0	1	5	4	0	**25**
YPS606	14	2	0	0	9	2	0	**27**
NCYC110	15	2	2	0	8	1	0	**28**
DBVPG6765	13	3	0	0	8	4	0	**28**
DBVPG6044	15	2	1	1	9	1	0	**29**
SK1	16	3	0	0	7	3	0	**29**
UWOPS87-2421	14	4	0	0	11	1	0	**30**
322134S	6	12	0	2	5	5	0	**30**
Y9	8	10	1	3	6	4	1	**33**
Y55	15	7	1	0	9	2	0	**34**
273614N	4	15	2	1	6	6	0	**34**
Y12	9	11	1	4	6	5	0	**36**
378604X	0	20	0	4	1	11	0	**36**
DBVPG6040	0	27	0	2	0	11	0	**40**
K11	23	2	5	0	9	2	0	**41**
YS9	1	27	0	4	1	8	0	**41**
YIIc17_E5	7	18	0	4	6	6	0	**41**
UWOPS05-227-2	24	7	0	0	11	0	0	**42**
UWOPS05-217-3	27	3	0	0	7	6	0	**43**
UWOPS83-787-3	8	21	0	1	6	7	0	**43**
UWOPS03-461-4	29	0	0	0	15	0	0	**44**
NCYC361	0	27	0	4	0	13	0	**44**
YS4	9	24	1	4	6	5	0	**49**
DBVPG1853	14	18	1	5	2	9	0	**49**
	**339**	**311**	**15**	**42**	**215**	**145**	**1**	**1068**
